# A scalable metabolite supplementation strategy against antibiotic resistant pathogen *Chromobacterium violaceum* induced by NAD^+^/NADH^+^ imbalance

**DOI:** 10.1186/s12918-017-0427-z

**Published:** 2017-04-26

**Authors:** Deepanwita Banerjee, Dharmeshkumar Parmar, Nivedita Bhattacharya, Avinash D. Ghanate, Venkateswarlu Panchagnula, Anu Raghunathan

**Affiliations:** Chemical Engineering Division, CSIR-National Chemical Laboratory, Pune, India

**Keywords:** Antibiotic resistance, Metabolomic, Flux balance analysis, Flux variability analysis, Redox homeostasis, NAD, NADH, Metabolism

## Abstract

**Background:**

The leading edge of the global problem of antibiotic resistance necessitates novel therapeutic strategies. This study develops a novel systems biology driven approach for killing antibiotic resistant pathogens using benign metabolites.

**Results:**

Controlled laboratory evolutions established chloramphenicol and streptomycin resistant pathogens of *Chromobacterium*. These resistant pathogens showed higher growth rates and required higher lethal doses of antibiotic. Growth and viability testing identified malate, maleate, succinate, pyruvate and oxoadipate as resensitising agents for antibiotic therapy. Resistant genes were catalogued through whole genome sequencing. Intracellular metabolomic profiling identified violacein as a potential biomarker for resistance. The temporal variance of metabolites captured the linearized dynamics around the steady state and correlated to growth rate. A constraints-based flux balance model of the core metabolism was used to predict the metabolic basis of antibiotic susceptibility and resistance.

**Conclusions:**

The model predicts electron imbalance and skewed NAD/NADH ratios as a result of antibiotics – chloramphenicol and streptomycin. The resistant pathogen rewired its metabolic networks to compensate for disruption of redox homeostasis. We foresee the utility of such scalable workflows in identifying metabolites for clinical isolates as inevitable solutions to mitigate antibiotic resistance.

**Electronic supplementary material:**

The online version of this article (doi:10.1186/s12918-017-0427-z) contains supplementary material, which is available to authorized users.

## Background

A post-antibiotic apocalypse portends the utility of antibiotics being massively compromised through evolution of antibiotic resistance. The genetic basis of several antibiotic resistant populations have been delineated via whole genome sequencing to the level of identifying resistance genes [1–4]. Antibiotic resistance genes catalogued through these efforts have rarely provided individualized therapies. Penicillin resistance in *Streptococcus pneumoniae* is a consequence of mutations in putative iron transport systems [1]. Stress pathways contribute to glycopeptide resistance in *Staphylococcus aureus* [5]. The evolution of antibiotic resistance in pathogens is characterized by uncontrolled proliferation even in the presence of drugs. Growth and energy generation are two principal dimensions of cell function and proliferation. This duality of cell function, orchestrated by metabolic networks, is critical for survival and governs resistance. Redox homeostasis is important to effectively harness reducing power produced through the catabolism of various substrates and to utilize this power in the anabolism of cellular components such as DNA, lipids and proteins. Metabolic regulation and gene expression modulation are now recognized as major players in antibiotic resistance [6–9]. β-Lactamases are amongst the most common causes of bacterial resistance to β-Lactam antimicrobial agents [10]. Derepression of secondary metabolism in *Nocardiopsis*, identified through metabolome characterization, was a consequence of acquired resistance [9]. In *E. coli* it was reported that overflow metabolism and reactive oxygen species (ROS) formation are inherent cellular response to antibiotic lethality [11]. A recent study implicates accelerating cellular respiration rates in the bactericidal mode of action [2]. Specific metabolites have been associated with varying degrees of killing antibiotic tolerant pathogens (persisters) by stimulating proton motive force (PMF) and increasing antibiotic uptake [12, 13]. Promoting Tricarboxylic acid cycle (TCA)/Krebs cycle by glucose/alanine activation that subsequently also increase PMF stimulating uptake of antibiotic have initiated death in multi-drug resistant *Edwardisiella tarda* [13]. Stoichiometric flux balance models based on evolutionary optimality predict outcomes of single environment evolution accurately and can compute operational flux states, growth and energy phenotypes of resistant/susceptible cells [14–18]. Investigating altered metabolism and connecting to evolved resistant genotypes may provide simple strategies to overcome drug resistance and induce susceptibility to existing antibiotics.

**Fig. 1:**
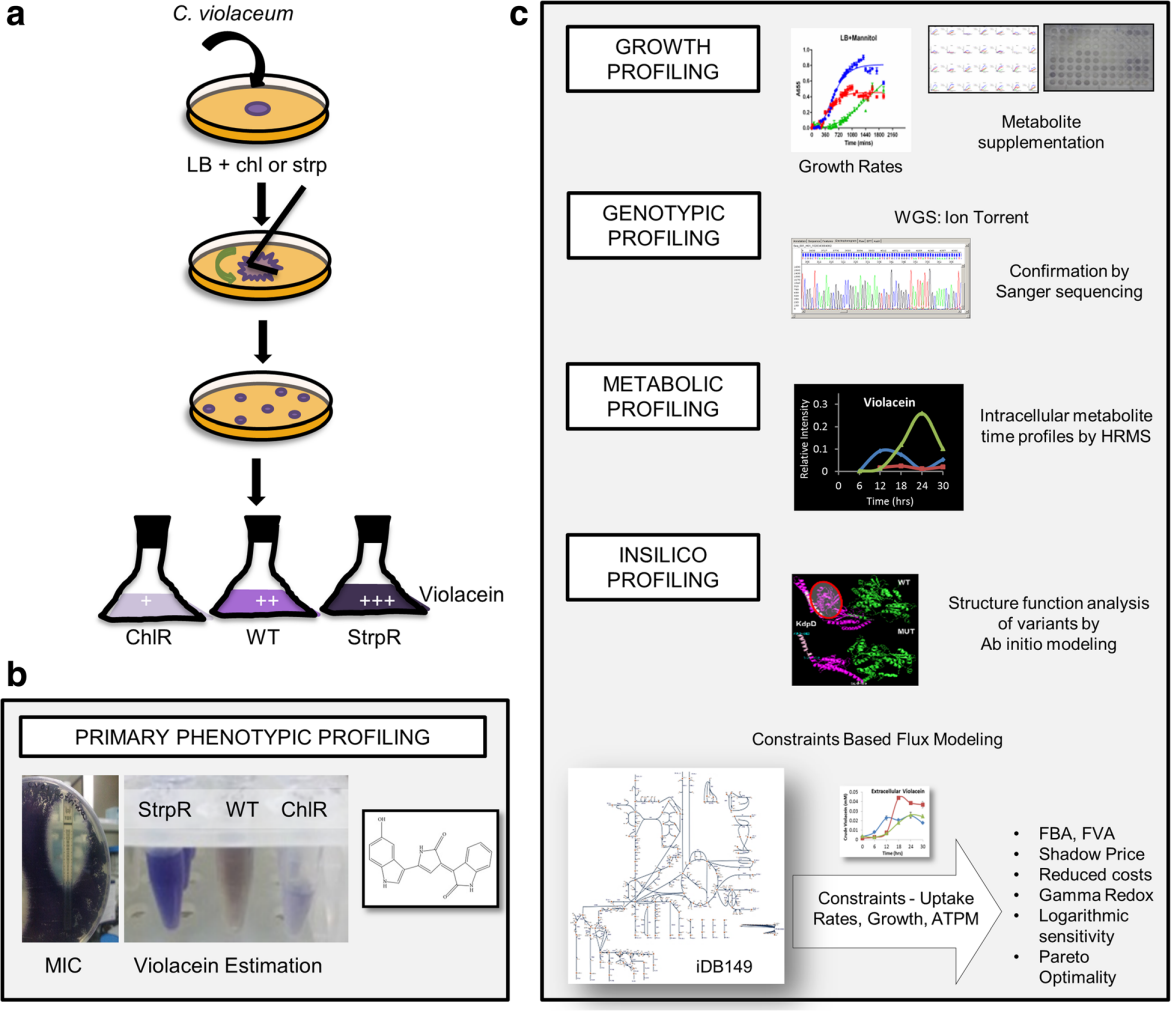
Schematic/work flow of experimental design – From evolution to emergence. **a** Adaptive laboratory evolution (ALE) of antibiotic resistant populations of C. violaceum under sub-lethal concentrations of antibiotic. 5 μl of overnight culture of C. violaceum was evenly spread onto LB agar plates with respective antibiotic (10 μg/ml) and were incubated at 30 °C until colonies appeared on the agar plates. After clonal purification the resistant populations were cultured and showed characteristic violet color pigment, violacein. ChlR shows lower intensity of pigmentation while StrpR showed higher levels as compared to WT. **b** Primary phenotypic profiling performed to confirm the evolution of resistance against the two antibiotics using minimum inhibitory concentration (MIC) and violacein estimation (refer Methods for details). **c** Systems Biology approach used in this study with basic growth profiling, metabolite supplementation experiments, genotypic profiling using whole genome sequencing (WGS), HRMS metabolomics, and *in silico* structural analysis of variants and flux balance modeling using iDB149 network with constraints derived from in house data. This scalable pipeline allows understanding the genotype-phenotype relationship of the resistant pathogens

In this study, we have identified benign metabolites to stimulate antibiotic action and death of streptomycin and chloramphenicol resistant pathogen *Chromobacterium violaceum*. Primarily a zoonotic pathogen, it is opportunistic in humans and converts the essential amino acid tryptophan to violacein, a blue-violet pigment [19]. The oxidative dimerization of two tryptophan molecules is an essential and regulatory step for the synthesis of violacein scaffold [20–22]. *Chromobacterium violaceum* is also reported sensitive to aminoglycosides, chloramphenicol, and tetracycline and resistant to ampicillin, penicillin, and first-generation cephalosporins [23, 24]. Our work highlights for the first time that differential violacein levels could act as a potential biomarker for resistance to two distinct classes of antibiotics. Here we pioneer a scalable workflow (Fig. 1) from controlled evolution of resistance to rationally identifying metabolites that induce drug susceptibility using systems biology approaches. Constraints-based flux balance modeling, being an integral part of systems biology approach, was used for the first time to predict that disruption of redox homeostasis was causal for antibiotic action. Compensatory metabolic reprogramming to overcome redox cofactor imbalance was delineated. In addition the *in silico* resistant growth phenotype was predicted to be a function of the rigidity of flux network.

## Methods

### Adaptive Laboratory Evolution (ALE)

The detailed ALE workflow is well described in Fig. 1a. *Chromobacterium violaceum* strain ATCC 12472^T^, wild type, (*C. violaceum* or WT) was obtained from the American Type Culture Collection Center (ATCC), USA. The wild type *C. violaceum* was routinely cultured on Luria-Broth (LB, Hi-Media-M575) and maintained at 30 °C with continuous aeration in a shaker incubator set at 180 revolutions per minute (rpm). *C. violaceum* was tested to be susceptible to low concentrations of both antibiotics (Chloramphenicol: MIC 8 μg/mL and Streptomycin: MIC 10 μg/mL in liquid culture and 60 μg/mL in agar plates). Antibiotic resistant strains of *C. violaceum* were evolved separately under controlled laboratory environments using the two antibiotics, chloramphenicol (chl) and streptomycin (str) at sub-lethal concentrations (10 μg/mL) on Luria Bertani agar (LBA) plates. Clonal purification by repeated sub-culturing of the colonies obtained on LBA plates with antibiotic (10 μg/mL) resulted in single colonies. These colonies were cultured in LB with antibiotic (10 μg/mL) and further cryopreserved in 50% glycerol and all genotyping and phenotyping experiments done by thawing the frozen vials and sub-culturing in LB with antibiotic (10 μg/mL) at 30 °C with continuous aeration in a shaker incubator set at 180 rpm until mentioned otherwise (Fig. 1d).

### Minimum Inhibitory Concentration (MIC) determination

**Fig. 2:**
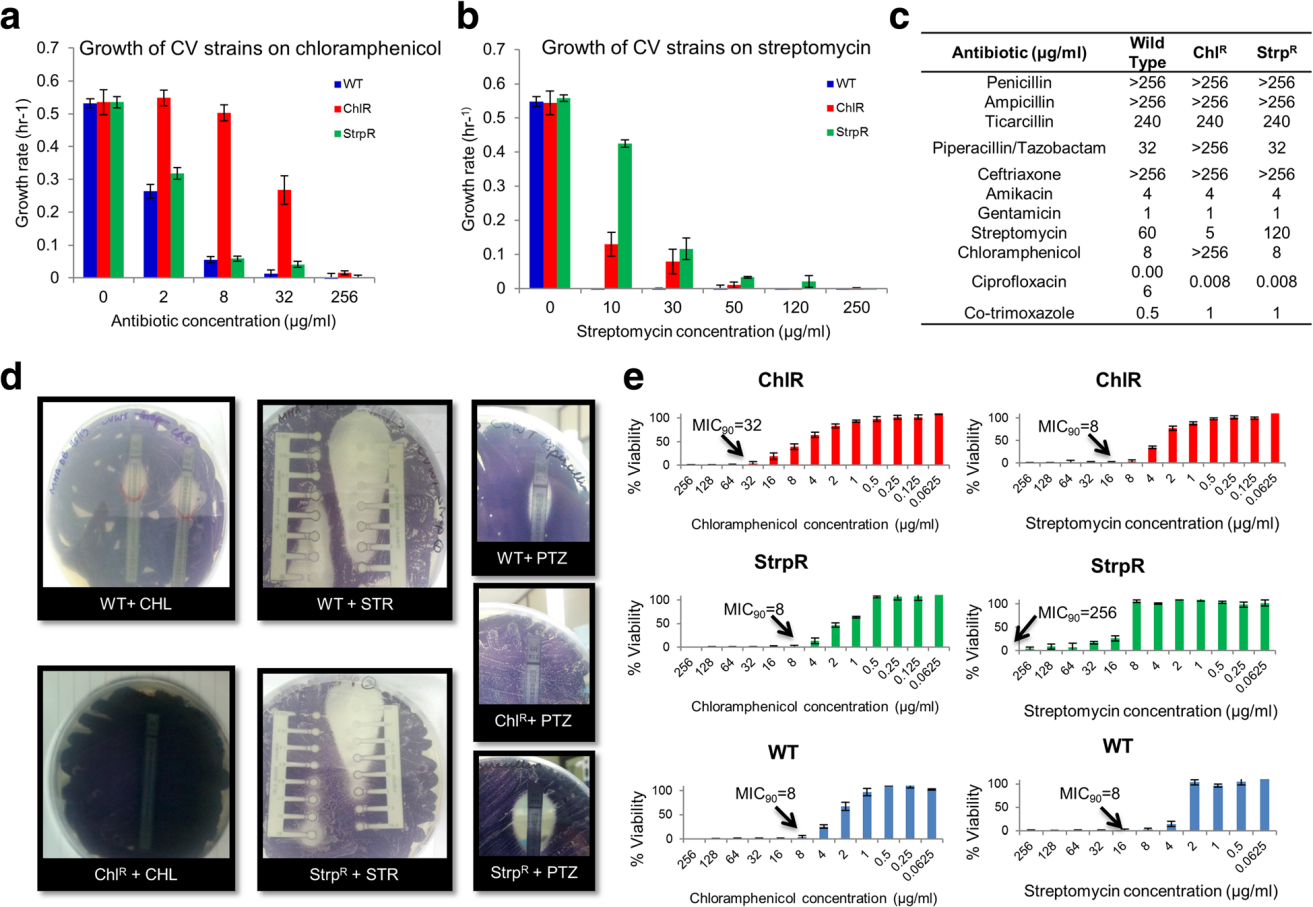
The evolved phenotypes of antibiotic resistance: Growth profiling and minimum inhibitory concentration typing across sensitive and resistant populations. **a** Growth rate on varying concentration of chloramphenicol showing a 17 and 7 fold change relative to wild type at 32 μg/mL for ChlR and StrpR respectively. **b** Growth rate on varying concentration of streptomycin showed no growth for WT and ChlR at 30 μg/mL and considerable growth rate for StrpR. **c** MIC using EzyMIC^TM^ Strips for 11 antibiotics. **d** Mueller Hinton agar plates showing primary resistance against chloramphenicol (Chl) and streptomycin (Str) and secondary resistance developed against piperacillin/tazobactam (PTZ). ChlR shows no zone of inhibition contrary to an elliptical zone of inhibition in case of WT and StrpR. **e** Broth dilution method shows high MIC values for the resistant populations against the respective antibiotics that they were evolved on. Legends are Blue for WT, Red for ChlR and Green for StrpR. Means ± S.D. represented in a, b and e (n ≥ 3)

Antibiotic susceptibilities were determined with EzyMIC^TM^ Strips (HiMedia Laboratories, India) on Müller-Hinton agar plates using the manufacturer’s instructions (Fig. 2c and d). The MICs were further determined using broth micro-dilution method for *C. violaceum* (at 30 °C, 180 rpm) according to the dilution method described previously [25] (Fig. 2e).

### Effect of varying concentration of antibiotics on growth profiles and growth rate estimation

All strains were profiled for growth by varying concentrations of antibiotics at 30 °C, 180 rpm. Exponential-phase cultures were prepared at 30 °C, 180 rpm using a shaker incubator and further used to inoculate 3 mL of LB to an initial OD_600_ of 0.1. Antibiotic stock solutions were added to yield desired concentrations of the antibiotic ranging from 0 to 256 μg/mL. Cultures were incubated in a shaker incubator at 30 °C, 180 rpm and bacterial cell densities were estimated hourly using a spectrophotometer. Growth profile assays for each *C. violaceum* strain were performed in triplicate using independent starter cultures and antibiotic stocks. Growth rate was estimated graphically from growth curves by plotting the natural log values of OD_600_ for each time point and determining the slope by linear regression. A minimum of four time points were used to determine the growth rate (Fig. 2a and b).

### Growth profiling on different exogenous carbon and nitrogen sources

**Fig. 3:**
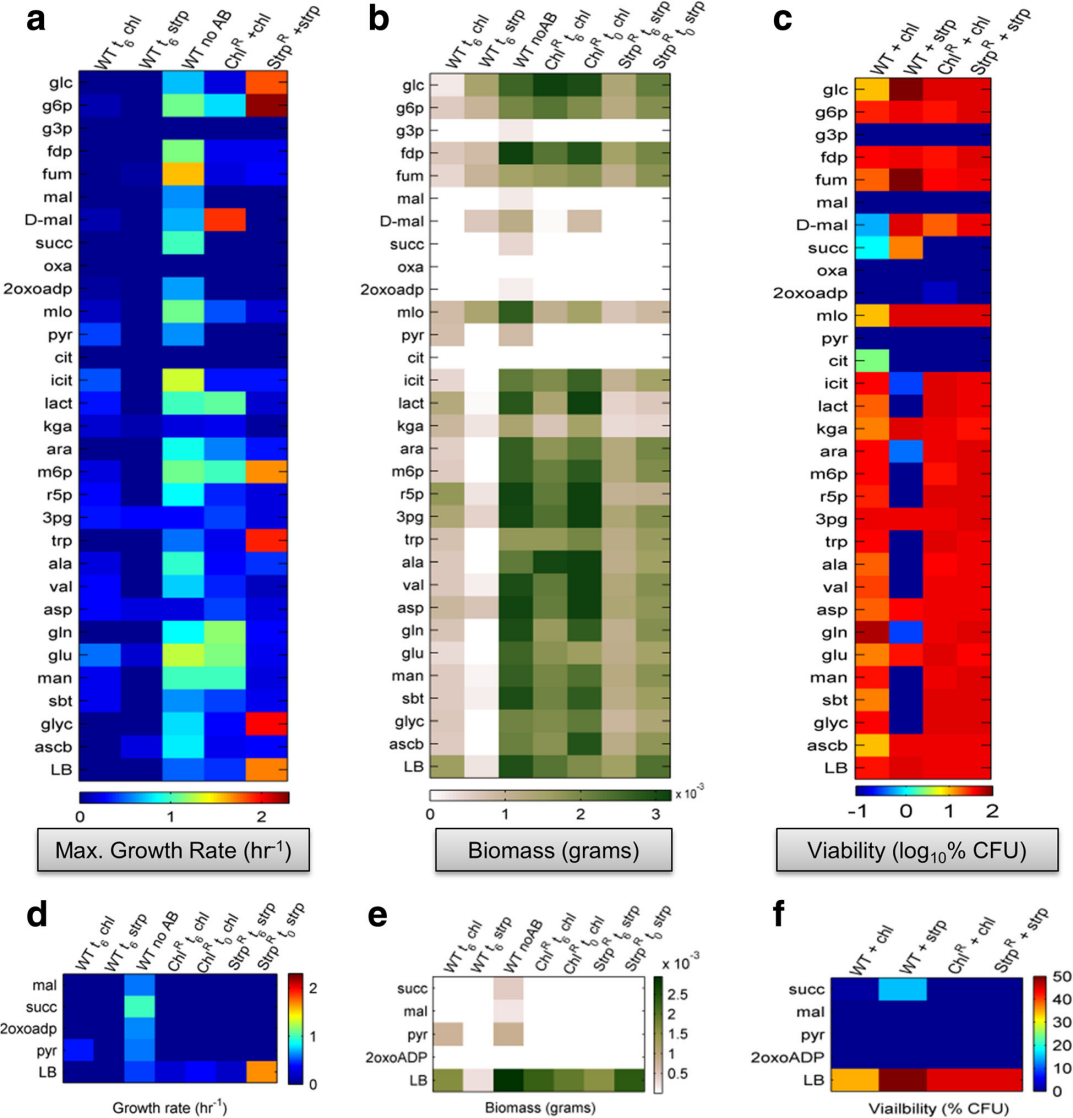
Systematic evaluation of microenvironment metabolite supplemented antibiotic effects on biomass, growth and viability for the three populations. **a** The heat map represents the exponential growth rates (measured fitness) of the WT, ChlR and StrpR populations on multiple microenvironment metabolites. The predominantly blue-scale of the wild type in presence of antibiotics (first two columns) indicate the bactericidal and bacteriostatic effect of antibiotics. The last two columns show the evolution of resistance as indicated by the increased growth rates. The Inset (**d**) highlights the four metabolites maleate, succinate, pyruvate and 2oxoadipate on which growth rates are minimal even for the resistant populations. **b** The heat map represents the maximum amount of biomass after 30 h (as cell dry weight) that is produced by the WT, ChlR and StrpR populations on multiple microenvironment metabolites. The Inset (**e**) highlights the four metabolites maleate, succinate, pyruvate and 2oxoadipate on which biomass was minimal. The effect of initial colony forming units was assessed by adding at the start of the culture (t0) and 6 h after growth (t6). **c** The heat map represents viability (as log 10 values of colony forming units/ml) after 48 h in the absence of antibiotics on rich LB media plates. The inset (**f**) once again confirms the effect of the four metabolites maleate, succinate, pyruvate and oxoadipate on which viability is null

*C. violaceum* was cultured overnight, then diluted to a density of 0.002 (Optical density at 600 nm, OD_600_), mixed with an equal volume of LB that contains different metabolites at a final concentration of 2 mg/mL, except for lactate which was at a concentration of 0.27 mg/mL. *C. violaceum* was cultured in 96-well flat-bottom plastic microplates at 30 °C for 30 h with or without antibiotic pressure. 30 different metabolites were tested in biological triplicates. The plates were monitored for growth, biomass and violacein using iMark™ Microplate Absorbance Reader (BIO-RAD) at 550 nm and 655 nm at regular time intervals. The amount of violacein and dry cell weight were determined using 550 nm and 655 nm readings and compared to standard calibration graphs (Additional file 1: Figure S4) for quantitation purpose. Different conditions were tested wherein one set had the respective antibiotics, to which the strains were resistant, from zeroth time point (t0) and in the other set, antibiotic was added 6 h (t6) post inoculation. After 30 h the t6 set of plates were used to plate fresh LBA plates without any antibiotic to analyze viable colonies after the 30 h incubation period. Growth rates were measured for the overall 30 h duration experiment in four different phases: pre-6 h phase, post 6 h phase, overall growth rate and a maximum growth rate. Same phase wise analysis for biomass and violacein was also performed. Curve fitting, visualization and analysis of the different plots for this experiment were done using GraphPad Prism Version 6.01 (GraphPad Software, San Diego California USA, www.graphpad.com). Nonlinear curve fitting was done using Gompertz growth equation [26] for the growth data points (Additional files 2, 3 and 4). All the heat maps were generated using MATLAB platform (Fig. 3).

### Whole genome sequencing

In order to extract genomic DNA for whole genome re-sequencing, ChlR and StrpR cultures were revived from previously cryopreserved glycerol stocks on LBA plates with respective antibiotic at 30 °C. These cultures have been previously tested for all the phenotypic traits as described in the results section. A single colony from LBA was cultured in LB broth at 30 °C, 180 rpm and mid – log phase cells were harvested for genomic DNA extraction using the DNeasy Blood and Tissue Kit (Qiagen, USA) according to the manufacturer’s instructions. The quality of the genomic DNA was assessed for RNA contamination using A260/A280 ratio and visualized on agarose gel. The DNA was also quantitated using Qubit before library preparation. Genome sequences were obtained for the two evolved populations, ChlR and StrpR, using Ion Torrent PGM^TM^ (Life Technologies) NGS using the 314^TM^ chip with mean read length of 180 and 188 base pairs respectively. The sequencing data analysis for ChlR and StrpR samples showed 86 and 84% of the bases read were of ≥ Q20 quality respectively. For 77.07% of ChlR and 71.3% of StrpR samples the genome base coverage was 20x and for 18.61 and 10.01% coverage was 100x. The assemblies and comparative analysis against NCBI sequence of *C. violaceum* (accession number NC_005085) were also performed. The genome sequence data for this publication has been deposited at Sequence Read Archive (SRA) (http://www.ncbi.nlm.nih.gov/sra) under accession number SRP072862.

### Confirmation of mutations by Sanger Sequencing

All NGS identified sequence variations were confirmed by Sanger sequencing. Primers (Additional file 1: Table S5) were designed to amplify around 200 to 600 base pairs (bp) amplicons such that the nucleotide of interest was at a position for easy read during Sanger sequencing. Amplification and sequencing was performed by Eurofins Genomics India Pvt. Ltd.

### *In silico* Structure - Function analysis of the mutation acquired

**Fig. 4:**
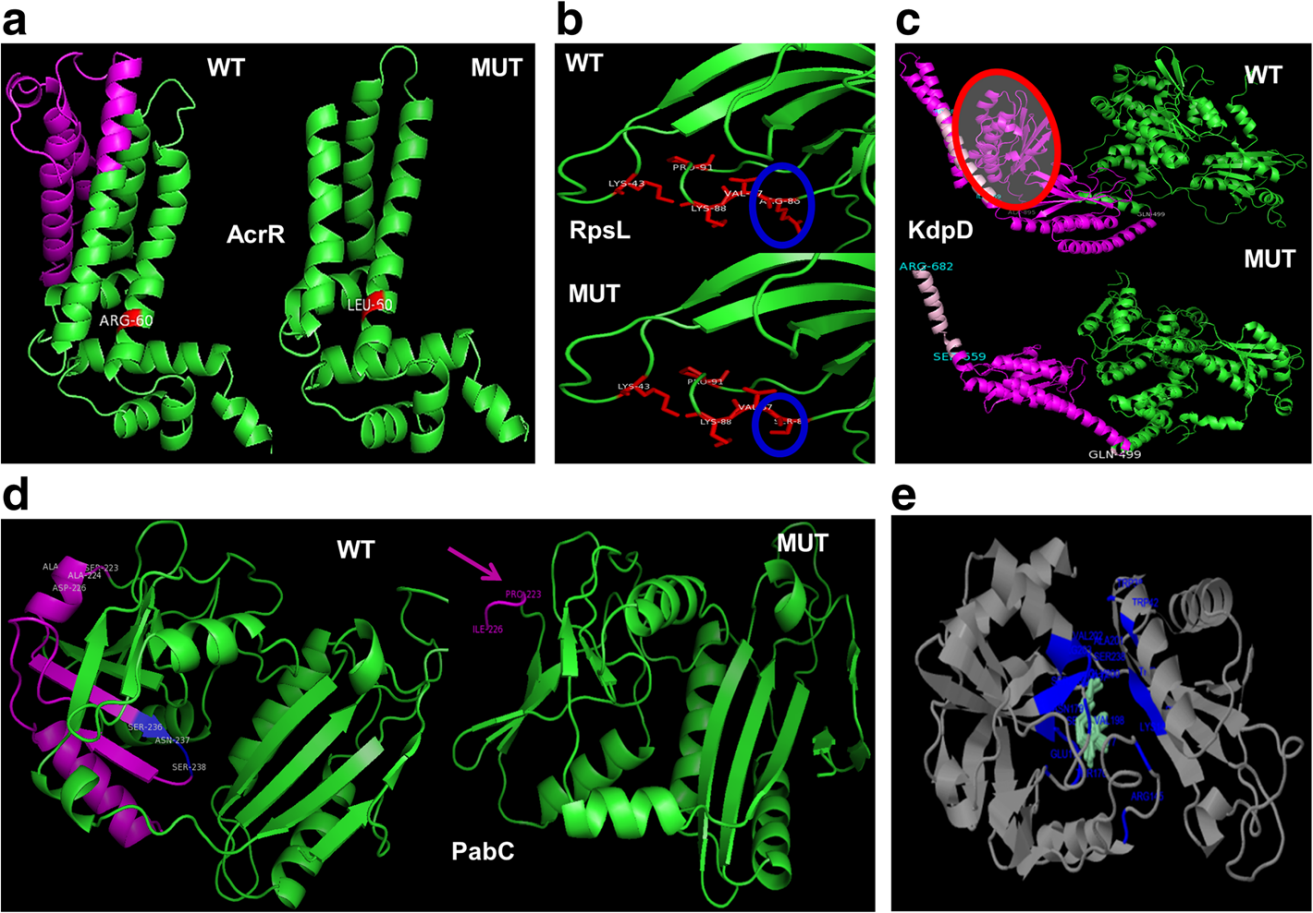
*In silico* protein structure and function alterations due to altered genotypes confirmed by sanger sequencing. **a- d** Ab-initio models for wild type (WT) and mutant (MUT) proteins for AcrR, KdpD and PabC using ROBETTA and homology model for RpsL. **e** 3DLigandSite representation of the ligand binding residues (blue) including Ser238 among others, lost in the mutated variant of *pabC* gene as shown in (**d**)

Ab-initio models were made using ROBETTA server [27] (http://robetta.bakerlab.org) except for RpsL (homology modeling was used) and the models generated were checked for various parameters for model assessment, such as Ramachandran Plots using PROCHECK. Finally, visualization and manipulation of the three dimensional models were performed using the software PyMOL (Schrödinger, LLC, 2015). In addition 3DLigandSite [28] was used to get a better understanding of the structure-function change post mutation/variation (Fig. 4).

### Preparation of intracellular metabolite extracts

For performing metabolomics experiments, the three different populations of *C. violaceum* were inoculated (10% inoculum of overnight starter culture) and incubated in a shaker incubator at 30 °C, 180 rpm for 30 h. 2 mL of cell cultures were harvested at the end of 0, 6, 12, 18, 24 and 30 h by centrifugation at 12000 g at 4 °C for removal of extracellular media. The following steps were carried out on ice. The pellets were reconstituted with ice-cold ethanol for quenching as well as maximal extraction of metabolite features as used in previous study [29, 30]. For collecting the extracts, the suspension was centrifuged at 14000 g at 4 °C. The intracellular extracts were aliquoted (100 μL) and immediately stored at −80 °C till further use.

### Spectrophotometric analysis

The intracellular as well as extracellular extracts were used for violacein estimation with BioPhotometer (Eppendorf) by comparing to standard calibration graphs for quantitation purpose as previously described [31].

### LC-MS analysis of intracellular metabolites

The intracellular extracts were dried in centrivap (Labconco) at 4 °C, followed by reconstitution in 100 μL of 10% water in acetonitrile containing 4 μM atorvastatin (internal standard). 10 μL of each sample was pooled to prepare a technical quality control (QC) sample. Metabolic profiling of samples was carried out on High Performance Liquid Chromatography – Heated Electrospray Ion Source – High-resolution Mass spectrometry (HPLC-HESI-HRMS). The separation was achieved on Sequent (Zwitter ionic hydrophilic interaction Liquid Chromatography) ZIC-HILIC column (100 mm*2.1 mm*5 μm, Merck Millipore) column using HPLC consisting of Accela quaternary gradient pump, a degasser and Accela autosampler (Thermo Fisher). The column was maintained at 45 °C using column oven (PerkinElmer). The mobile phase for elution consisted of 0.1% formic acid in deionized water (Mobile phase ‘A’) and 0.08% formic acid in acetonitrile (Mobile phase ‘B’). Gradient was set with 5% of mobile phase A (0–5.0 min, 300 μL/min), 13% A (15.0, 300 μl/min), 45% A (20.0 min, 300 μL/min), 90% A (23.0 min-25.0 min, 300 μL/min), 5% A (27.0–32.0 min, 700 μL/min). Heated electrospray ionization (HESI) source was used as an interface between Liquid Chromatography (LC) and High-resolution Mass Spectrometry (HRMS) instruments. The spray voltage of the source was set at 3.7 kV with capillary temperature 300 °C, sheath gas 45 units, auxiliary gas 10 units, heater temperature 390 °C and S-lens RF at 50 units. The data was acquired in range of 70–1050 *m/z* at resolution of 70,000 Full Width at Half Maximum (FWHM) with Automated Gain Control (AGC) target 1e6 and injection time of 120 ms. Two technical replicates, each of 5 μL sample volumes were injected during analysis in both positive and negative ion mode. A total of 7 QC samples were run at beginning, intermittently and end of the run.

### LC-MS Data analysis

For data analysis, the Qual browser module of Xcalibur (Thermo) was used for manual inspection of presence of metabolite of interest through accurate mass - extracted ion chromatogram (AM-XIC). A mass extraction window (MEW) of 20 ppm around monoisotopic *m/z* of possible adduct was used to generate the XIC. After establishing the retention time (Rt) and peak width of respective metabolites as well as internal standard (IS), Tandem Mass Spectrometry (MS/MS) was carried out in respective ion modes to confirm their identities. The MS/MS data was analyzed using fragment search tool in METLIN [32] data base (http://metlin.scripps.edu) and Mass frontier 7.0. A data processing method from processing setup module of Xcalibur software was prepared to integrate and generate area under peak data. The peak areas of metabolites were normalized to the area response of uniformly spiked internal standard, atorvastatin (ATV). This ratio is representative of the intracellular metabolite concentration (abundance) after taking into consideration the dilution factor for each sample extract. Principal component analysis (PCA) was used to get an overview of the data and to reduce the high dimension of the data set. For each metabolite, we calculated the temporal variation, using the coefficient of variation (CV):

$$ \mathrm{C}\mathrm{V} = \sigma /\mu, $$

where σ and μ are the standard deviation and mean of the measurements across the time points respectively.

### AP-MALDI of intracellular metabolites

It was also estimated on Thermo Q-Exactive mass spectrometer (MS) coupled with an atmospheric pressure - matrix-assisted laser desorption/ionization (AP-MALDI) source equipped with a solid state Nd:YAG laser operating at 355 nm. A mixture of 2,5-dihydroxybenzoic acid (2,5-DHB) and 2-cyano-4-hydroxycinnamic acid (CHCA) was used as a matrix for the analysis. Samples were mixed with internal standard (2,4-diamino-6-methyl-1,3,5-triazine) before spotting on MALDI target plate in 6 replicates. The instrument was operated in full MS scan mode within *m/z* 50–750 at resolution 35,000 FWHM. Spray voltage at 2.5 kV, capillary temperature at 250 °C, AGC target of 1e6 and 500 μs injection time were optimized before beginning analysis. Laser fluence was optimized at 70% and Pulse Dynamic Focusing (PDF) value of 100 μs was used with automated rastering motion chosen for data acquisition.

### AP-MALDI data analysis

Data analysis was performed with Thermo XCalibur Qual Browser, mMass [33] for qualification and in-house software MQ v 5.0 (http://www.ldi-ms.com/services/software) for quantification [34] with a chosen MEW of 20 ppm.

### Constraints based modeling of *C. violaceum* central metabolism: network reconstruction

Stoichiometric network analysis based on the constraint-based modeling framework has been proven to be a valuable tool to study cellular metabolism and phenotypic capabilities of many organisms [35–38]. -The small-scale central metabolic model of *C. violaceum* presented here is a manually curated stoichiometric network reconstruction and model that allows probing special characteristics of this bacterium. It was done using available literature data [39–42] as well as information from databases such as KEGG, Biocyc, Metacyc. Further the violacein biosynthesis reaction list, including reaction stoichiometry, reversibility, sub-cellular localization, and gene locus/loci for each reaction comprising core metabolism were also incorporated into the model. The biomass equation for the model was also modified in order to take into account for tryptophan contribution towards biomass production. For all simulations maximization of the biomass equation was fixed as the objective function until mentioned otherwise. The detailed description of the model can be found in Additional file 5. The model was initially validated using a set of 10 substrate utilization BIOLOG GN2 plate data existing in literature [43–45].

Further a set of constraints that define the antibiotic susceptible WT and differentiated the evolved populations (ChlR and StrpR) were determined. The constraints used in different simulations included (i) Substrate (Glucose) uptake rates (ii) Growth yields (iii) Violacein secretion (iv) ATP maintenance costs associated with molar growth yields of each strain as discussed (Table 3, Additional file 5). The specific growth rates were calculated using 1 g biomass as the basis. The growth yields thus calculated were compared across the three strains. The goal of the simulations was to understand the flux distribution *in silico* and the sensitivity of growth yield to various precursors with specific reference to the cofactors NADH, NADPH and ATP. Constraints based flux balance analysis (FBA), as described in the following section, was used to simulate for growth (maximize biomass objective function) and violacein production. Constraints-based methods were used to perform a comparative analysis between the susceptible and resistant populations to understand the connections between metabolism and resistance.

Implementation of the central metabolic reconstruction for *C. violaceum* and constraints-based analysis was done using Constraints Based Reconstruction and Analysis (COBRA) Toolbox 2.0.2 [46] with MATLAB v 7.11, (R2010b) and TOMLAB/CPLEX v7.7 optimizer. MATLAB codes for all referenced COBRA functions are available at the COBRA’s website (https://opencobra.github.io/).

### Flux Balance Analysis (FBA) and associated sensitivity parameters

Flux-balance analysis is a method for assessing the systemic properties and cell behaviors of a metabolic genotype. In short the primal FBA problem, equation (1) describes the steady-state mass balances of the biochemical reaction network [47–50]

$$ \mathrm{Maximize}\ \mathrm{Z}={\mathrm{c}}^{\mathrm{T}}\mathrm{v} $$

1 $$ \mathrm{Subject}\ \mathrm{t}\mathrm{o}\ \mathrm{S}.\mathrm{v}=0 $$

$$ {\mathrm{v}}^{\mathrm{LB}}\ge \mathrm{v}\ge {\mathrm{v}}^{\mathrm{UB}} $$

where c, v, v^LB^, and v^UB^ are vectors of length n, and S is the m x n stoichiometric matrix.

Mathematically, the S matrix acts as a linear transformation between the vector that defines fluxes through n reactions in the biochemical network and the vector of the time derivatives of the concentrations of m metabolites involved in these reactions. The fundamentals of FBA have been widely and critically reviewed [46, 51–54].

The function optimizeCbModel(model), in COBRA toolbox was used for maximization of pathogen growth or biomass by fixing the objective function to be the biomass equation in the model.

Two sensitivity parameters – shadow prices and reduced costs [46] - were assessed in order to understand the effects of changing biomass, ATPM, metabolites and reactions of the different populations of *C. violaceum*. Shadow price corresponds to the sensitivity of the growth rate as an objective function (Z) in response to a change in the availability of a metabolite (i), and indicates how much an increment in that metabolite will increase or decrease the growth rate. Analogous to shadow price, reduced cost is the sensitivity of the objective function in response to change in fluxes of a particular reaction and its effect on the objective. In addition to the primal solution (optimal fluxes), the LP solver provides the corresponding dual solution i.e., shadow price and reduced cost for the FBA problem.

Scaled reduced costs [55] and logarithmic sensitivity coefficient [56] were also calculated as shown in equation (2) and (3) which better assess the sensitivity taking into account the substrate and growth yield. The logarithmic sensitivity coefficient (D_i_) represents the sensitivity of each precursor yield to its biosynthetic demand whereas scaled reduced costs (W_i_) are used to assess the limiting reactions.

2 $$ {\mathrm{W}}_{\mathrm{i}} = \left({\mathrm{v}}_{\mathrm{i}}.{\mathrm{w}}_{\mathrm{i}}\right)/\mathrm{Z} $$

3 $$ {\mathrm{D}}_{\mathrm{i}}=\mathrm{d}\mathrm{X}/\mathrm{d}\mathrm{M}.\mathrm{d}\mathrm{M} $$

In equation (2) v_i_ is the flux through a particular reaction having a w_i_ reduced cost associated with it. Z is the objective function value, in this case being biomass. Similarly, in equation (3) dX/dM is the associated shadow price to a particular metabolite and dM is the coefficient of the metabolite in the objective function equation.

Another sensitivity parameter, gamma redox, the difference between the shadow prices for the redox couplet (NADH/NAD) and (NADPH/NADP) were calculated [51].

4 $$ {\upgamma}_{{\mathrm{redox}}_{\left(\mathrm{NADH}/\mathrm{NAD}\right)}}={\upgamma}_{{}_{\mathrm{NAD}\mathrm{H}}}-{\upgamma}_{{}_{\mathrm{NAD}}} $$

5 $$ {\upgamma}_{{\mathrm{redox}}_{\left(\mathrm{NADPH}/\mathrm{NADP}\right)}} = {\upgamma}_{{}_{\mathrm{NADP}\mathrm{H}}}-{\upgamma}_{{}_{\mathrm{NADP}}} $$

This is an index of available reducing capacity available to the cell and whether it is limiting or in excess for biomass formation. A positive shadow price suggests available reducing capacity in excess of the optimal demand for growth and where it is negative, limiting for optimal growth. A positive shadow price doesn’t necessarily indicate accumulation but also suggests metabolic rewiring into overflow metabolism.

### Flux Variability Analysis (FVA)

FVA was utilized to investigate the resulting space of feasible flux distributions [57]. FVA can be set up in COBRA toolbox using the function fluxVariability(). One can thus determine the minimum and maximum flux value that each reaction in the model can take up while satisfying all constraints on the system for a specific objective. The objectives under consideration for this study include optimal growth. These will be considered as forced or fixed fluxes. Differences in these unique forced fixed rates in resistant populations as compared to wild type indicate metabolic reprogramming. To highlight the differences between the antibiotic sensitive and resistant populations, we classified reactions in the network based on their minimum and maximum flux values and assigned categories that reflect their rigidity or flexibility. Nine categories can thus be mapped onto the flux variability map based on the magnitude and direction of the flux (Additional file 1: Table S4a) ranging from category 1 for forward direction (positive fixed) fixed flux, i.e., minimum and maximum flux values were same, a non-zero positive value to category 9 wherein the minimum and maximum flux was zero (blocked). Specific attention was paid to reaction rates that were uniquely determined (i.e., if upper and lower boundaries as computed by FVA coincide; Categories 1 and 4). Changes between such rigid fluxes (1 and 4) to more variable flux capabilities (2,3,5,6,7 and 8) would reprogram the metabolic network by either changing the direction of equilibrium or modulating magnitude and span of the reaction rates.

### Dynamic Flux Balance Analysis (dFBA)

dFBA was utilized to qualitatively predict the outcomes of growth in batch culture conditions matching our experimental condition [38]. The resistant populations ChlR and StrpR needed to be assessed for the onset of overflow metabolism and secretion patterns as observed in FVA simulations. The dFBA can be set up in COBRA toolbox using the function dynamicFBA() [58]. It is an implicit iterative process wherein at each iteration; FBA is used to simulate for growth, nutrient uptake and by-product secretion rates using an initial concentration for nutrients, which are in turn used to calculate biomass and nutrient concentrations in the culture at the end of the step. The same values are used to calculate maximum uptake rates of nutrients for the next time step.

### Glucose measurements

Glucose levels of *C. violaceum* cultures (WT, ChlR and StrpR) were measured using MyQubit Amplex Red Glucose Assay Kit (Thermo Fischer Scientific) according to the manufacturer’s instructions using Qubit 2.0 Fluorometer (Life Technologies, CA, USA). The assay is based on the D-glucose dependent generation of hydrogen peroxide with glucose oxidase (GO) followed by the horse radish peroxidase (HRP) catalyzed oxidation of colorless, stable Amplex Red (10-acetyl-3,7-dihydroxyphenoxazine) to fluorescent resorufin.

### NADH and NAD measurements

NAD/NADH levels of *C. violaceum* cultures (WT, ChlR and StrpR) were measured using a commercially available kit (MAK037, Sigma Chemical, St. Louis, MO, United States) according to the manufacturer’s instructions (Fig. 6c to e). NAD total (NAD and NADH) or NADH levels are quantified in a colorimetric assay at 450 nm using iMark™ Microplate Absorbance Reader (BIO-RAD).

## Results

### Adaptive Laboratory Evolution (ALE)

The schema for the workflow followed in this study (Fig. 1) involved the evolution of *Chromobacterium violaceum* strain ATCC 12472^T^ (*C. violaceum* or WT) from a small inoculum onto Luria Bertani agar (LBA) plates chloramphenicol (chl) and streptomycin (str), both targeting protein translation at the ribosomal subunit level. To provide strong evolutionary pressure while maintaining a sizeable population, the concentration of antibiotic was chosen such that no more than 60% of growth was inhibited. Once defined this sub-lethal concentration (SLC), of the antibiotic (10 μg/mL) was not varied throughout the evolution as well as other experiments unless specified. The adaptive evolution continued for about 3 weeks and when the first positive trait appeared, these colonies were cultured multiple times on LB agar plates with the respective antibiotics followed by colony purification. Multiple clones that were evolved in parallel were colony purified. Broth cultures of the respective antibiotic resistant population were used for Minimum Inhibitory concentrations (MIC) calculations. One of the parallel lines of evolved clones resistant to antibiotics was further used in genotypic, phenotypic and metabolic profiling studies (Fig. 1d).

### Evolution of antibiotic resistance and fitness

We first analyzed that growth rates and kinetic profiles of the resistant populations support evolution towards fitness on both antibiotics. The effect of varying antibiotic concentration on growth rate was studied (Fig. 2a and b). The growth rates of the chloramphenicol resistant (ChlR) population was reduced to 50% at 32 μg/ml chloramphenicol, while the streptomycin resistant population (StrpR) growth rate was lowered down to just 15% of that without antibiotic. At even 10 μg/ml of streptomycin the growth rate of the StrpR population was reduced by only 10%. The growth rate exponentially decreased with increasing concentration of the antibiotic in the wild type and evolved resistant populations (ChlR and StrpR). The resistant populations improved in growth rate and biomass yield substantially even in the presence of higher concentrations of antibiotic in contrast to the wild type (WT). Surprisingly, no fitness costs associated with the acquired resistance were observed in the absence of antibiotics.

Next, we assessed broad-spectrum antibiotic susceptibility for ChlR and StrpR populations via estimation of MIC on 11 different antibiotics (Fig. 2c). The population evolved on chloramphenicol, ChlR, had an MIC much greater than the wild type, being able to resist high titres of 256 μg/ml of chloramphenicol. The streptomycin evolved population, StrpR was able to resist twice the amount of antibiotic as the wild type reaching a titre of 120 μg/ml. StrpR populations showed low MICs for chloramphenicol indicating higher sensitivity. Only the ChlR population showed cross-resistance to Piperacillin/Tazobactam (PTZ) combination (Fig. 2d). Similar trends were observed when MIC values were estimated by broth dilution method (Fig. 2e) for chloramphenicol (>256 μg/mL) and streptomycin (120 μg/mL) and were represented as percentage viability of the cells.

### Effect of exogenous metabolites and antibiotics on growth

A systematic evaluation of benign microenvironment metabolites in excess of being limiting (Additional file 1: Table S2) showed unique fitness landscapes and associated costs for the evolved and wild type populations (Fig. 3). Wild type *C. violaceum* (WT) does not show capacity to utilize citrate, oxalate and glyceraldehyde-3-phosphate (Additional files 2, 3 and 4). Streptomycin (bactericidal) showed a more profound effect on growth, unable to support growth on 50% of the substrates tested while chloramphenicol (bacteriostatic) affected growth on only 7/30 (23%) of the substrates (Table 1 and Fig. 3a, c). The ChlR and StrpR populations showed fitness costs associated with growth on 13 and 17 substrates respectively. StrpR populations showed lag for extended period of times on many substrates (consistent with mutations discussed in the next section). Lowered fitness is observed on glycolytic intermediates like fructose-1,6-diphosphate and other TCA cycle intermediates. Strikingly, the ChlR population recorded almost no growth and viability on organic acids maleate (C3), pyruvate (C3), succinate (C4) and 2-oxoadipate (C6) even in the presence of chloramphenicol antibiotic (Fig. 3b and d). The StrpR population exhibited similar growth patterns with the only exception of D-malate (C3) also being able to make the resistant population susceptible again to antibiotic.

**Table 1:** Fitness costs of antibiotic resistance on multiple substrates

	Growth Rates (hr^−1^)					Time Lag (hr)				
Metabolites	WT + chl	WT + str	WT	ChlR	StrpR	WT + chl	WT + str	WT	ChlR	StrpR
Glucose	**0.00**	**0.00**	0.71	0.21	1.80			3	9.5	2
Glucose-6-phosphate	0.09	0.01	1.08	0.77	2.23	3	3	2	3.5	2
Glyceraldehyde 3-phosphate	**0.00**	**0.00**	**0.00**	**0.00**	**0.00**					
Fructose 1,6-bisphosphate	**0.00**	0.01	1.14	0.23	0.25		3	1.5	6	10
Fumarate	**0.00**	0.06	1.55	0.21	0.27		3	1.5	12	13.5
Maleic acid	**0** ^**d**^	**0.00**	0.60	**0** ^**a**^	**0** ^**c**^			1		
D-Malic acid	0.08	0.01	0.67	1.89	**0** ^**b**^	2.5	15.5	1.5	0	
Succinate	**0.00**	**0.00**	1.00	**0** ^**a**^	**0** ^**b**^			2.5		
Oxalic acid	**0.00**	**0.00**	**0.00**	**0.00**	**0.00**					
Oxoadipic acid	0.04	**0.00**	0.62	**0** ^**e**^	**0** ^**c**^	0		0		
Malonic acid	0.11	0.03	1.08	0.45	0.17	2.5	2.5	1.5	2	18
Pyruvate	0.41	**0.00**	0.58	**0** ^**e**^	**0** ^**b**^	4		2.5		
Citric acid	**0.00**	**0.00**	**0.00**	**0.00**	**0.00**					
Isocitric Acid	0.46	**0.00**	1.31	0.32	0.32	10		3.5	3.5	12.5
L-Lactic acid	0.29	**0.00**	0.99	1.05	0.16	3		4.5	4.5	18
Ketoglutaric acid	0.15	0.09	0.19	0.24	0.04	0	0	3.5	0	21
L-Arabinose	**0.00**	**0.00**	0.88	0.54	0.29			3.5	3.5	13.5
Manose 6-Phosphate	0.18	**0.00**	1.08	0.98	1.68	3		2.5	3.5	2.5
Ribose 5-phosphate	0.28	**0.00**	0.86	0.33	0.18	2.5		2.5	6	6
3-phosphoglyceric acid	0.29	0.26	0.28	0.40	0.18	2	2	0	6	6
L-Tryptophan	**0.00**	**0.00**	0.53	0.23	1.92			2	2	2
L-Alanine	0.21	**0.00**	0.96	0.28	0.36	2.5		2.5	6	3.5
L-Valine	0.28	**0.00**	0.74	0.33	0.14	2		2	6	6
L-Aspartate	0.27	0.20	0.19	0.41	0.18	2.5	2.5	0	6	10
L-Glutamine	**0.00**	**0.00**	0.83	1.15	0.26			2.5	3.5	13
L-Glutamate	0.52	0.16	1.28	1.12	0.23	3	3	3	3	12.5
Mannitol	0.24	**0.00**	1.00	0.98	0.20	3		2	3.5	8.5
D-Sorbitol	0.24	**0.00**	0.58	0.40	0.25	12.5		2.5	6	9.5
Glycerol	**0.00**	**0.00**	0.78	0.28	1.99			2.5	9.5	2.5
L-Ascorbic acid	**0.00**	0.18	0.81	0.23	0.27		3	3	6	12
Luria bertani	**0.00**	**0.00**	0.47	0.37	1.69	3	3	1.5	3	3

Altered kinetic parameters represented as Growth Rates and Time lag for the three populations of *C. violaceum* (WT, ChlR and StrpR) on multiple micro-environment metabolites. Zero growth rates are represented in bold

^a^t6 initial till 180 min

^b^t0 initial till 180 min

^c^t0 first 60 min

^d^3 to 18 h growth

^e^growth after 24 h

### Altered genotypes and *in silico* protein functional analysis in resistance

In order to see how genotype was shaping the growth phenotype of the resistant populations, we re-sequenced the whole genome and confirmed 14 sequence changes (Table 2, Additional file 1: Table S5) using capillary sequencing. The sequence changes were seen to affect the protein structure and function *in silico* as discussed (Fig. 4).

**Table 2:** Summary of variants confirmed using Sanger sequencing post whole genome sequencing

S.N.	Gene locus	Gene name	Nucleotide change	Type	Amino acid change	GENE STRETCH	Gene detail
1	CV_0436	*acrR*	G179T^a^	SNP	R60L	456,438 - > 457,085, 648 bp/215 AA	Transcription repressor of multidrug efflux pump
			G375GAT^a^	INS	Premature termination, 141 AA		acrAB operon, TetR (AcrR) family
2	CV_4365	*marC*	C4648G	SNP	No Change	708,719 - > 4,709,366	multiple drug resistance protein
3	CV_4191	*rpsL*	G117T^b^	SNP	R86S	4,519,516 < − 4,519,887, 372 bp/123 AA	30S ribosomal protein S12
4	CV_3410	*pabC*	A147deletion^b^	DEL	Premature termination, 226 AA	3,703,561 < − 3,704,373, 813 bp/270 AA	4-amino-4-deoxychorismate lyase
5	CV_1596	*kdpD*	G167deletion^b^	DEL	Premature termination, 682 AA	1,719,330 < − 1,722,017, 2688 bp/895 AA	2 component regulatory protein sensor kinase. Osmosensitive K+ channel histidine kinase KdpD (EC 2.7.3.)
6	CV_0066	CV_0066	G655C^c^	SNP	P219A	75,121 < − 76,422, 1302 bp/433 AA	Hypothetical protein
7	Nt CDS	nt cds	1263524^c^	SNP		between CV_1197 and tRNA Ser	Cv_1197 - polysaccharide/polyol phosphate ABC transporter ATPase
8	CV_0464	CV_0464	A4273C^c^ G4274A^c^ T4276A^c^ C4277G^c^ G4278C^c^ C4344G^c^	SNP SNP SNP SNP SNP SNP	Synonymous	478,148 - > 483,712, 5565 bp/1854 AA	Hypothetical protein, Homologous to Fibronectin type III domain protein

Out of 57 genes and 25 non coding (Nt CDS) obtained after whole genome sequencing using Ion Torrent platform only eight of them were confirmed using capillary electrophoresis of which some were unique or common to the resistant populations

^a^variant confirmed in ChlR

^b^variant confirmed in StrpR

^c^variant confirmed in both the strains

The ChlR population acquired mutations in *marC* [59] and the transcription repressor *acrR* of the tripartite AcrAB-TolC multidrug efflux pump [60–62]. Mutations in *marC* were silent substitutions. The repressor protein suffered premature truncation after translation of 141 amino acids and a non-synonymous substitution R60L resulting in altered domain that is involved in ligand binding for repression (Fig. 4a and Additional file 1: Figure S5c). In case of the StrpR population, a non-synonymous substitution R86S was detected in *rpsL,* coding for the 30S ribosomal protein S12 [63–66] along with deleterious mutations in *kdpD*, encoding for the sensor kinase of the two-component signal transduction system (TCS) [67] and *pabC,* encoding the pyridoxyl 5’ phosphate (PLP)–dependent enzyme 4-amino-4-deoxychorismate lyase catalyzing PABA biosynthesis [68, 69]. *Ab initio* models built using ROBETTA and ligand binding analysis using 3DLigandSite showed that the mutated KdpD and PabC proteins truncated after 682 and 226 amino acids respectively resulting in loss of critical ligand binding or sensory domains (see Discussion).

### The metabolic phenotype of resistant populations

**Fig. 5:**
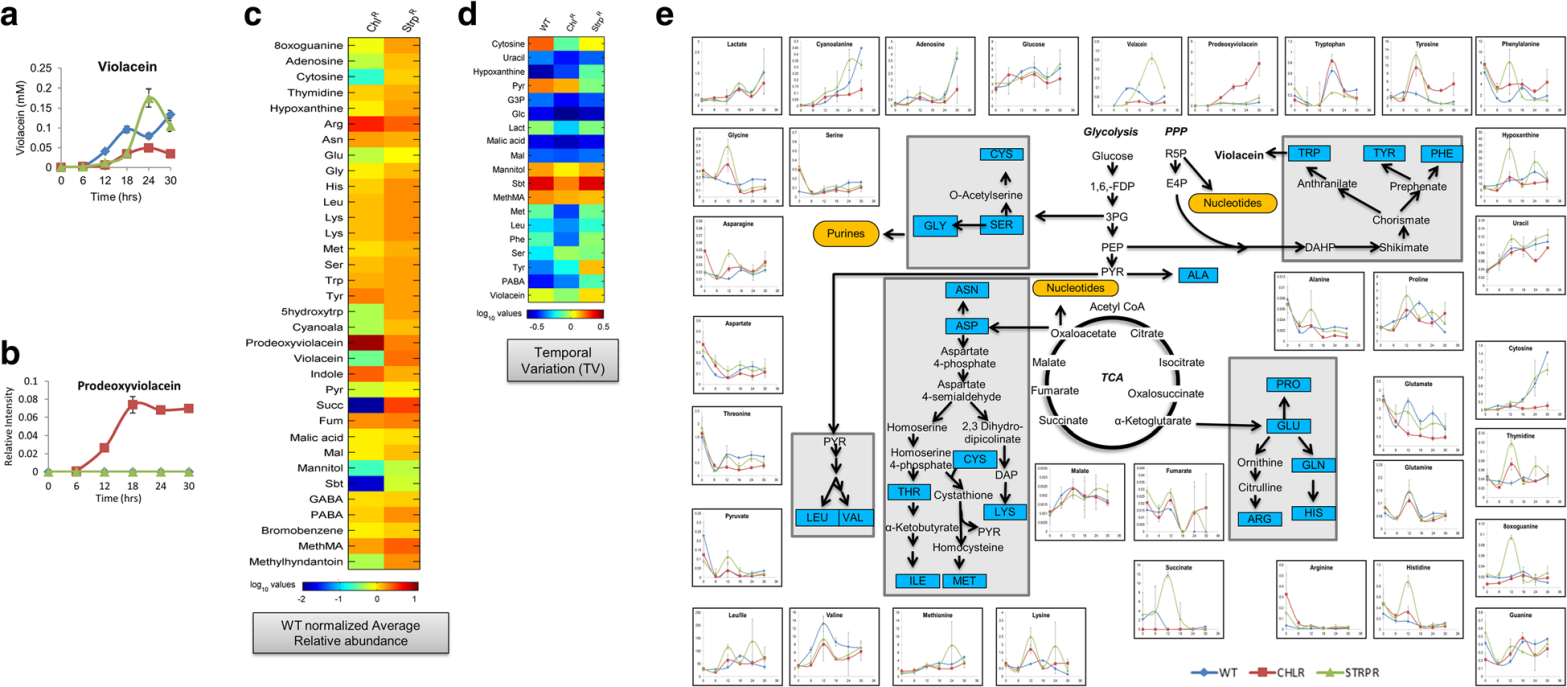
The metabolic basis of antibiotic resistance through dynamic metabolomic profiling shows metabolic reprogramming. **a** Violacein with its differential abundances as compared to wild type in the StrpR (50% increase) and ChlR populations (50% reduction). **b** Prodeoxyviolacein measured only in ChlR population. **c** Fold change with reference to wild type population across resistant populations in their average intracellular relative abundance (log 10 values). **d** Temporal variation of metabolite abundances across sensitive and resistant populations (log 10 values). **e** The oscillatory or linear behavior with varying amplitude, period and phase lag during growth on glucose across sensitive and resistant populations. The Central Carbon Metabolism Network is drawn for quick correlation. *Solid blue squares* show all amino acids, Fructose-1,6-biphosphate (1,6-FDP), D-ribose-5-phosphate (R5P), D-erythrose-4-phosphate (E4P), glycerate-3P (3PG), phosphoenolpyruvate (PEP), pyruvate (PYR). *Yellow rounded rectangles* show nucleotides. Various metabolite time profiles for the three strains are shown. All the values were normalized to the internal standard (Refer Methods for details). Graph legends: *Blue* – WT, *Red* – ChlR, *Green* – StrpR. Means ± S.D. represented in (**a,b** and **e**) (n ≥ 2)

To determine the fundamental genotype-phenotype relationship and establish a metabolic basis for antibiotic resistance, intracellular metabolomic profiles were measured using Liquid Chromatography – High-resolution Mass Spectrometry with Tandem Mass spectrometry (LC-HRMS with MS/MS) (Fig. 5, Additional file 6). Including both ion modes, total 126 metabolites were screened. At the end of the analysis, 59 metabolites were finally selected based upon qualification criteria of mass accuracy, MS/MS confirmation, elution profile, reproducibility of response of technical QC samples and biological relevance. The details of these 59 metabolites are mentioned in Additional file 6. Relative abundance was calculated by normalizing metabolite peak area response with that of internal standard (metabolite of interest peak area/internal standard peak area). This ratio is representative of the intracellular metabolite concentration (abundance) after taking into consideration the dilution factor for each sample extract across the three different populations of *C. violaceum*. Metabolite relative abundance levels in the three populations span three orders of magnitude intracellularly (Additional file 1: Figure S1). Guanosine, methylmalonate, glutamine and aspartate vary slightly, but high order of magnitude differences are seen in PABA, succinate, leucine, hypoxanthine and violacein. To validate that the metabolism is different in the wild type (WT) and resistant strains (ChlR and StrpR), Principal Component Analysis (PCA) of quantitative features of metabolites of intermediary metabolism extracted from LC-HRMS data was performed (Additional file 1: Figure S2). Score plots of principal components for both biological replicates, show trends that showcase maximum separation of data with respect to different time points (PC1) and also clustering of data points based on susceptibility or resistance to antibiotic (PC3). Differential expression of features exhibited by StrpR was captured by PC3 in both the biological replicates. A significant difference after 6 h time point in scores of StrpR shows a distinct deviation in metabolic behavior in comparison to the other populations studied. This is also observed in the growth profiles of StrpR. The ChlR strain shows separation from the WT and the StrpR populations in terms of intermediary core metabolism on glucose as also identified through flux balance modeling discussed in following sections. The significant metabolites after almost 30 h of growth include deoxyviolacein, xanthine and β-cyanoalanine while the metabolites in the early hours of growth include more core metabolite candidates like fumarate, maleate, malate, succinate and pyruvate (Additional file 1: Figure S2). The metabolite abundances also show oscillatory behavior with varying amplitude, period and phase lag (Fig. 5e). Leucine, lysine and proline had a characteristic oscillatory behavior with a period of 12 h; also there is phase lag in WT compared to resistant populations. Certain metabolites such as arginine and adenosine showed negligible changes. Intermediate of violacein biosynthesis pathway, prodeoxyviolacein was seen to increase linearly only in ChlR whereas it was very low for the other two i.e., WT and StrpR. The presence of prodeoxyviolacein, a precursor only in ChlR (Fig. 5b) potentially explains the lowered violacein through limited availability of cofactor NADPH since tryptophan levels are similar. Increased recycling of nucleotides through salvage pathways was reflected in high levels of adenosine, xanthine and hypoxanthine in the resistant populations (Fig. 5c and e). 8-oxoguanine, a major oxidized base lesion formed by reactive oxygen species, was higher in the StrpR population indicating potential oxygen radical effects.

The linearized dynamics around the steady-state level of metabolites is captured by the temporal variation (Fig. 5d, See Methods). Growth limiting metabolites are known to show relatively lower temporal variation [17]. Temporal variation (TV) identified potential growth limitation by malate, glucose, glyceraldehyde-3-phosphate and uracil across all populations. Phenylalanine and methionine are potentially growth limiting (low TV) in ChlR while pyruvate only in StrpR. Tyrosine and serine are less growth limiting (high TV) in the resistant populations than the wild type. Pyruvate and malate showed low average intracellular concentrations or had low temporal variation in the two resistant populations (Fig. 5c and d).

### *In silico* prediction of NAD/NADH balance and redox homeostasis

**Fig. 6:**
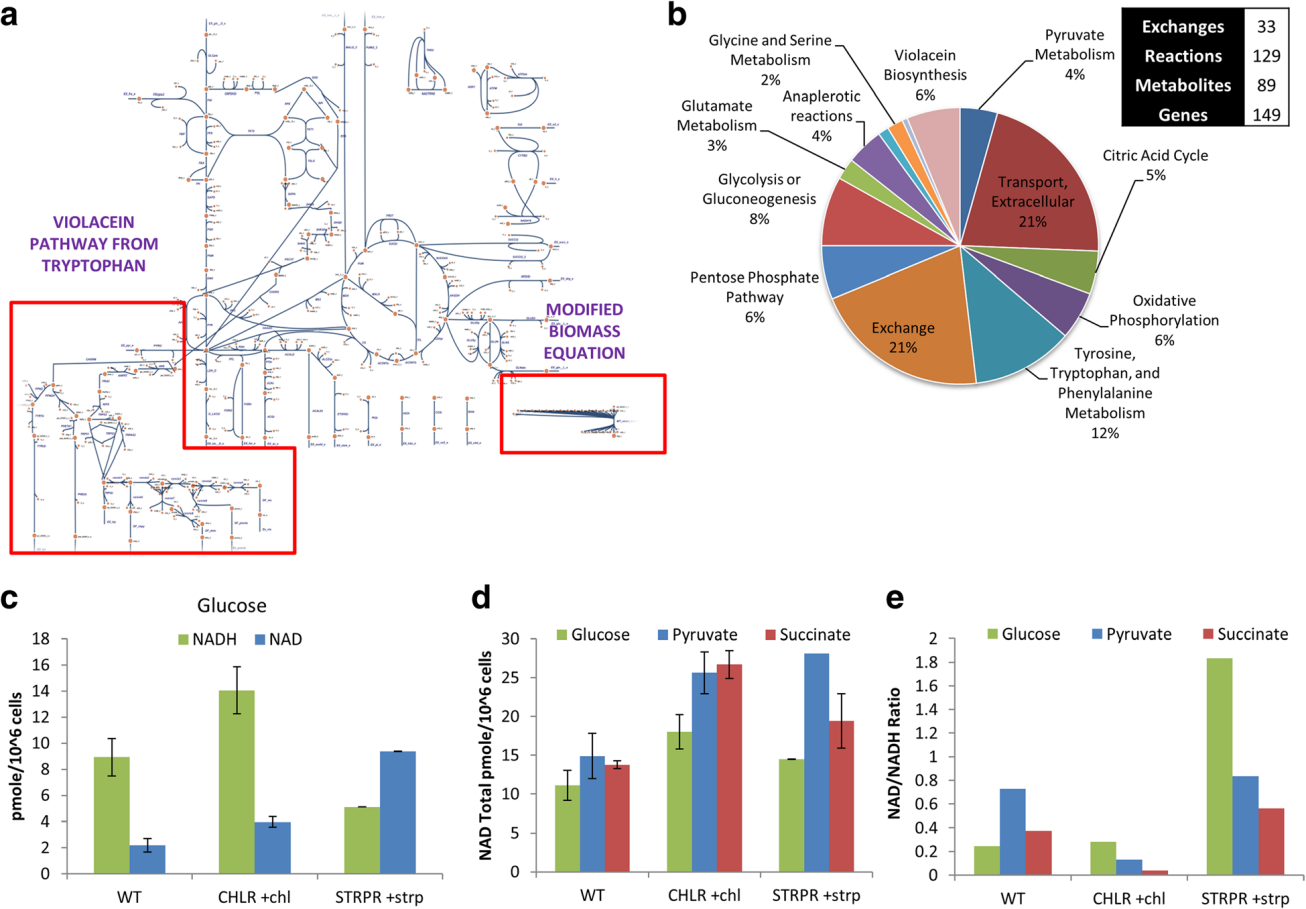
Constraints-based Modeling predicts disruption of redox homeostasis and rewiring of metabolic network for compensation. **a** Core network representation of *C. violaceum* metabolism (iDB149) with tryptophan, violacein pathway (using Escher; https://escher.github.io/) and tailored biomass composition. **b** Reconstruction statistics and subsystem classification. **c - e** NADH and NAD experimental values attained for the three different strains using three different substrates – Glucose, Pyruvate and Succinate. Mean ± S.D. for triplicate samples represented

To delineate a metabolic basis for the emergent resistance, the heterogeneous components of resistant genomes and elucidated metabolic physiology were integrated into a constraints-based flux balance model. A central metabolic network reconstruction represented by the genotype of *C. violaceum* iDB149 was developed (Fig. 6a and b; Additional file 5). The network reconstruction represents the core metabolism for the pathogen including glycolysis, pentose phosphate pathway, TCA cycle, electron transport and basic amino acid metabolism. Although, primarily a generic reconstruction of central metabolism, it includes the virulence factor metabolism, tailoring it to mirror *C. violaceum* metabolism. It includes detailed amino acid metabolism of specifically tryptophan, due to its direct connection with the production of violacein, a virulence factor specific to this pathogen. A biomass equation was defined as a drain on metabolites present in intermediary metabolism and macromolecules in the precursor biomass based on *E. coli* and *Chromobacterium* legacy data. This biomass composition is kept constant throughout the analysis and between strains. In order to understand the effects of changing biomass composition, a logarithmic sensitivity coefficient [56] that represents the sensitivity of each precursor yield to its biosynthetic demand was calculated for each of the 14 precursors of biomass (Additional file 5). Rank ordering identified higher impact of cofactors NADH, NADPH and ATP to increasing growth. Molar growth yields calculated based on experimental glucose utilization data and the maximum growth yield for *Chromobacterium* (Additional file 5) was used to estimate the growth-rate independent energy ATP maintenance flux [38] (v_ATPM_) that represents the energy required to sustain basal cellular activities. The values interpolated/fitted from growth yield curves differed for the wild type and the resistant populations due to varying molar growth yields (Additional file 5, Table 3). The differential violacein phenotype (represented as a production/secretion rate constraint) calculated from experimental data was used to define resistant populations *in silico* (Additional file 1: Table S3). Based on the sensitivity analysis of violacein production and growth yields, a trade-off exists between the production of violacein and biomass production. Fixing this biosynthetic demand as a critical constraint in the model, growth rates (via growth yields) predicted for both resistant ChlR, StrpR and WT populations were consistent with experimental data. The experimental rates used for the simulations were from the exponential growth phase of the three different populations of *C. violaceum*. With these constraints determined by experiments, the wild type model was tested for prediction of carbon source utilization patterns using experimental legacy data [43–45] of Biolog™. The model being a core metabolic model was validated for utilization of carbon sources that had transporters included and growth against a small subset of substrates was accurate (Additional file 5). Further, the sensitivity of the yields to different biosynthetic demands, maintenance and changes in fluxes were probed. Shadow prices in the solution of the linear optimization problem of Flux Balance Analysis (FBA) define the sensitivity of the objective function with respect to each constraint indicating the utility of the metabolite in accelerating growth. Growth, as an objective in FBA, is defined as multiple simultaneous demands on precursors to make macromolecules related to biomass. In this context, a scaled shadow price for metabolites and scaled reduced costs for reactions that account for substrate and the growth yield are better sensitivity indicators (See Additional file 5 and Methods). The logarithmic sensitivity for cofactor NADH is the lowest in the ChlR populations followed by StrpR and differ from wild type at molar yields (Table 4). The 25% decrease in scaled shadow price values indicate that a compensation for that particular cofactor must have taken place during evolution resulting in a higher yield of that cofactor in the evolved strains for the already achieved higher growth and biomass yield. The logarithmic coefficients show that NADH and NADPH as compared to ATP may potentially play a role in increase in biomass yields through changing biomass composition. The reduced costs of each reaction indicate their significance in increasing the objective (growth). The alpha-keto glutarate dehydrogenase (AKGDH) reaction in the ChlR strain while the isocitrate lyase (ICL) reaction in the StrpR strain have scaled reduced costs associated with them (Table 4). Flux variability analysis (FVA) assesses the entire range of cellular function and the redundancy of optimal phenotypes. Applying FVA to identify reaction rates that can be uniquely determined allow us to explore the immutable or rigid metabolic state of the cell at maximal specific growth rate consistent with experimental data. Some reactions can be assigned fixed values, while the remaining calculable fluxes remain within the extreme bounds (Additional file 1: Table S4a). Uniquely computed reaction rates that are forced or fixed fluxes (coinciding upper and lower bounds) define metabolic rigidity and govern the plasticity of growth phenotype. Differences in these unique forced fixed rates in resistant and susceptible populations overall flux distribution indicate compensatory changes in metabolism due to antibiotic selection pressure. The constraints-based model identified two major features based on alternate optima predictions. Firstly, the resistant populations showed rigid flux distribution in secretion of overflow metabolites acetate and formate (Table 5). The onset of overflow metabolism and the details of secretion patterns were probed further using dynamic flux balance analysis (Additional file 1: Figure S3a - c). Both the resistant populations identified acetate as a common overflow metabolite. Dynamic FBA (dFBA) (Additional file 1: Figure S3a - c) qualitatively identified secretion of acetate and formate in that order in the ChlR population as indicated in the FVA and ethanol on lowering the oxygen uptake rates. The second major feature included reactions changing the rigid flux distribution in the wild type to a more flexible flux in the resistant populations. The reactions included alpha-keto glutarate dehydrogenase (AKGDH) and malate dehydrogenase (MDH) (Table 5). These change from a rigid flux configuration to more flexible one that potentially could modulate the direction and magnitude of flux involving NADH. The significance of alpha-keto glutarate (AKG) in the growth of the resistant populations is also supported by the high logarithmic sensitivity with respect to growth (Additional file 5). In order to probe this further, we looked at the scaled shadow prices, γ_redox_ and logarithmic sensitivity during experimental conditions (Additional file 1: Table S4b and Additional file 5) and identified NADH and NADPH to limit growth in the ChlR strain while ATP was also growth limiting in the StrpR strain. Thus, disruption of redox homeostasis through NADH/NAD ratios (Fig. 6, Table 5) and biomass precursor anabolism through NADPH/NADP ratios were identified as central to antibiotic action by FVA [57] and sensitivity/shadow price analysis [70]. A recent report uses a NADH oxidase enzyme system to delineate the role of NADH imbalance and show decoupling of electron transfer via ETC. and proton pumping for ATP synthesis [71]. Analysis of pareto fronts and trade-off between ATP and NADH and NADPH maintenance reactions (NADH/NADPH oxidases, not generally present in *Chromobacterium*) was performed to understand the modulation of NADH/NAD and NADPH/NADP ratios in growth. A reaction representing the NADH/NADPH oxidase (water forming) with the right balance of protons and oxygen was added to the model. This reaction essentially acts as a drain if there is excess NADH/NADPH in the system. On probing the relation to ATPase (representing ATP maintenance, ATPM) by a pareto front analysis (Additional file 1: Figure S3d - e, the differential relation for ChlR and StrpR was established. To reduce the growth of the ChlR strain to the wild type molar yield we identified NADH oxidase as a critical constraint (Pareto front analysis). For StrpR, however, both NADH and NADPH oxidases were needed. This is also suggested by γ_redox_ analysis (Additional file 1: Table S4b), which identified both NADH and NADPH as growth limiting in StrpR and only NADH as growth limiting in ChlR. At molar growth yields this suggests that excess NADH yields are indeed responsible for the excess growth associated with the resistance to chloramphenicol, while both NADH and NADPH yields play a role in StrpR. The rigidity of the flux held through AKGDH and MDH reactions was restored, when these constraints were added. Experiments confirmed an increase in NADH levels (Fig. 6e) in the ChlR population. For the StrpR population the NAD levels go up (Fig. 6e), seen in the molar yield simulations that show a 2 fold increase in flux through the NADH16 reaction that is the quinone associated conversion of NADH to NAD (Additional file 5).

**Table 3:** Constraints used in this study for simulation of growth for the three different populations of C. violaceum using iDB149

Model	Glucose uptake rate	Violacein secretion rate	Molar growth yield	ATPM	Biomass
WT	9.99	1.49	0.0312	6.24	0.23
ChlR	10.532	0.673	0.0314	9.74	0.327
StrpR	12.777	0.702	0.0504	5.69	0.644

Units for Glucose uptake rate and Violacein secretion rate are mmol/gDW/hr whereas hr^−1^ for Biomass and gDW/mmol of glucose for Molar growth yield

**Table 4:** Sensitivity parameters assessed using FBA - Scaled shadow prices, Logarithmic sensitivity and Maximum reduced costs

	Metabolite	Maximum Precursor Yield (M/Glc)	Shadow price in BOF (dX/dM)	Coefficient in BOF (dM)	Scaled shadow price (SSP)	Logarithmic sensitivity (LS)
WT	NADPH	0.004	−0.0079	13.028	−1.36E-04	−0.1024
	NADH	0.0061	−0.003	−3.547	−7.95E-05	0.0107
	ATP	0.0056	−0.0097	59.81	−2.37E-04	−0.5784
ChlR	NADPH	0.0034	−0.0054	13.028	−5.56E-05	−0.07
	NADH	0.0101	2.09E-18	−3.547	6.48E-20	−7.40E-18
	ATP	0.0034	−0.0108	59.81	−1.11E-04	−0.643
StrpR	NADPH	0.0009	−0.008	13.028	−1.11E-05	−0.1043
	NADH	0.0017	−0.0031	−3.547	−8.04E-06	0.0109
	ATP	0.0013	−0.0099	59.81	−1.93E-05	−0.5895
	Reaction ID	Reaction Name		WT	CHLR	STRPR
Maximum Scaled Reduced Cost	AKGDH	2-Oxoglutarate dehydrogenase		0	−8.11E-07	0
	EX_o2(e)	Oxygen Exchange		0	1.106	0.683
	ICL	Isocitrate lyase		0	0	−4.86E-07
	PGL	6 - Phosphogluconolactonase		0	−8.11E-07	−4.86E-07
	SUCCt3	Succinate transporter		0	−8.11E-07	−4.86E-07
	PPNDH	Prephenate dehydratase		0	−8.11E-07	0
	GLCt2	Glucose Transporter		0	−8.11E-07	−4.86E-07
	Rxnvio8	Reaction 8 of Violacein Synthesis		0	−6.56E-08	−2.81E-08

**Table 5:** FVA results showing category change in resistant strains as a function of antibiotic that involve redox cofactor balancing

Reaction ID	Reaction formula	WT	ChlR	StrpR
HEX1	atp[c] + glc-D[c] - > adp[c] + g6p[c] + h[c]	1	7	7
PYK	adp[c] + h[c] + pep[c] - > atp[c] + pyr[c]	7	1	1
AKGDH	akg[c] + coa[c] + nad[c] - > co2[c] + nadh[c] + succoa[c]	1	7	1^a^
MDH	mal-L[c] + nad[c] < = > h[c] + nadh[c] + oaa[c]	1	7	1^a^
FUM	fum[c] + h2o[c] < = > mal-L[c]	1	7	1^a^
SUCOAS	atp[c] + coa[c] + succ[c] < = > adp[c] + pi[c] + succoa[c]	4	7	4^a^
ACKr	ac[c] + atp[c] < = > actp[c] + adp[c]	7	4	4
PFL	coa[c] + pyr[c] - > accoa[c] + for[c]	7	1	7^b^
PTAr	accoa[c] + pi[c] < = > actp[c] + coa[c]	7	1	1
GLCt2	glc-D[e] + h[e] - > h[c] + glc-D[c]	1	7	7
ACt2r	ac[e] + h[e] < = > ac[c] + h[c]	7	4	4
FORti	for[c] - > for[e]	7	1	7^b^
EX_ac(e)	ac[e] < =>	7	1	1
EX_for(e)	for[e] < =>	7	1	7^b^

Increased TCA cycle/Oxidation Phosphorylation in the StrpR population and increased overflow metabolism population diverting from TCA cycle in the ChlR population. Of use here are Category definitions – 1 and 4 representing forced and fixed flux in either direction. 7 defined by negligible variable flux

^a^StrpR/WT flux fold is 0.52

^b^StrpR/WT flux fold is 0.32

## Discussion

The advent of genome-scale experimentation allows acquisition of heterogeneous data-types that are critical to delineating the genotype-phenotype relationship [1–4]. However, the mechanistic basis for the killing of antibiotic resistant populations only partially emerges through such data and requires integration of multiple data-types into a predictive scalable model. In this work we show that an integrative approach is able to predict the underlying mechanism related to redox and energy homeostasis operational at the level of cellular metabolism in *C. violaceum*. The model explains/predicts how benign metabolites in combination with antibiotics could potentially kill antibiotic resistant *Chromobacterium* populations by driving metabolism in a direction causing imbalance and disruption of the delicate redox or energy balance needed for the organism to survive.

Changed kinetic parameters (Table 1) of the two resistant populations in comparison to the susceptible wild type on several substrates indicated differential utilization and metabolic patterns resultant from altered genotypes and physiology via adaptive evolution of *C. violaceum*. Interestingly, fitness costs associated with the acquired resistance only manifested during growth on carbon sources and not in the environment used for evolution. The identification of four metabolites pyruvate, maleate, succinate and oxoadipate, potentially all electron donors, do not support growth across both the ChlR and StrpR resistant populations. The null post treatment viability count (Fig. 3e and f) make them ideal candidates for antibiotic therapy for resistant populations.

Also, these metabolites with the exception of maleate are electron donors and enter catabolism in central metabolism as glycolytic or TCA intermediates (Additional file 1: Figure S5). Each of the substrates is taken up and metabolized via specific dehydrogenases that involve the cofactor couple NAD/NADH. The results obtained highlight the importance of measuring fitness costs under multiple micro-environmental conditions. They provide a more relevant estimate of fitness in *Chromobacterium* and also reveal novel physiological weaknesses exploitable for drug development such as the redox homeostasis. Knowledge of such associated fitness costs in other pathogens can identify microenvironment metabolites that in combination with the antibiotic can target reduction of pathogen fitness.

The evolutionary fitness landscape of *Chromobacterium* can be viewed as a random adaptive (weighted) walk dictated by the drugs in the regime of strong selection and weak mutation (SSWM) [72, 73]. Such weighted walks in space of genotypes in the presence of chloramphenicol may have resulted in mutations in *acrR,* the transcription repressor of the tripartite AcrAB-TolC multidrug efflux pump and *marC* another multidrug efflux pump and may be potentially commutative. The continuous activity of the tripartite AcrAB-TolC multidrug efflux pump, which is proton dependent, could result in membrane potential changes due to efflux of small molecules like violacein. Antibiotics are known to activate the AcRAB-TolC pumps [62] and hence continuous de-repression may be a mechanism for evolution. The relatively few changes in genome sequence observed with chloramphenicol as selection pressure indicate a role beyond genetic causality in the resistant phenotypes.

Previously implicated mutations in *rpsL* (R86S; Fig. 4b) related to streptomycin resistance [63, 74] dictate an “error-restrictive”, hyper-accurate translation phenotype, accurate ribosomal function [65] and could explain the long lags and lowered growth rates observed for this population on specific carbon sources. Enhanced growth of the StrpR in poor carbon sources like glycerol probably occurs due to lower levels of the transcription factor, σS [75]. Lack of growth on pyruvate, succinate and maleate indicate potential induction of *rpoS* [76]. The mutated PabC protein involved in *de novo* folate biosynthesis via PABA could potentially lead to excess PLP known to perturb amino acid metabolism [77], including the observed tryptophan synthesis as observed in LC-HRMS with MS/MS profiles for StrpR intracellular temporal extracts. Streptomycin is known to cause leakage of low molecular weight antibiotic, ions and amino acids by damaging the permeability barrier creating potential secondary selection pressure [78]. This implies low cytoplasmic K+ could shape the evolution as a secondary selection pressure specifically based on the mutated cytoplasmic domain of KdpD TCS regulator. On genotypic profiling and protein functional analysis we reach a hypothesis that the antibiotic adapted landscape of *C. violaceum* with a few beneficial mutations and fitness distributions potentially supports the Gillespie [72, 73] landscape model for evolution of resistance. Whether these genome changes are causal or correlational need to be investigated.

Violacein with its differential abundances (Fig. 5a) as compared to wild type in the StrpR (>50% increase) and ChlR populations (~50% reduction) could be a potential biomarker for resistance. This could be potentially due to indole known to be shared in antibiotic resistant *E. coli* to provide cover for more susceptible bacteria [79]. *C. violaceum* accumulates tryptophan (downstream of indole) that is converted subsequently to virulence factor violacein. Potentially, developing a synthetic operon to convert tryptophan (found in all pathogens) to violacein may be useful as a visual reporter/biosensor of emergent resistance in even other infectious diseases. Reports exist that chloramphenicol lower intracellular indole and accumulation of tryptophan as observed [80]. Further exploration is needed to connect violacein to strategic intracellular communication and resistant growth phenotypes of the pathogen.

Using the *in silico* central metabolic network reconstruction representing *C. violaceum,* iDB149, we were able to understand emergent properties of redox and energy homeostasis across susceptible and resistant populations. On shadow price and reduced costs analysis of growth limiting metabolites and reactions, AKGDH and ICL were identified in ChlR and and StrpR respectively. The alpha-keto glutarate dehydrogenase (AKGDH) reaction is known to regulate oxidative phosphorylation, lysine and tryptophan synthesis. Isocitrate lyase (ICL) reaction on the other hand, has been implicated in pathogenesis and persistence in *Salmonella* and resistance in *Mycobacterium* [81]. The use of ICL involves shunting isocitrate through the glyoxylate shunt and bypassing part of the TCA cycle. This potential mechanistic difference between ChlR and StrpR in glucose metabolism is also evident in the intracellular metabolite profile (Additional file 1: Figure S2). AKGDH is also a known modulator for oxidative phosphorylation and dependent on the ADP/ATP ratios [82]. The flux through the ATP synthase is higher in the StrpR strain (Additional file 5) suggesting a potential increase in respiration rates as previously described [2]. The apparent lowering of ATP synthase flux potentially suggests a mechanism for decoupling of electron transfer from proton pumping in oxidative phosphorylation in the resistant populations. The StrpR population with mutations in *rpsL* may need a model of regulation and metabolic to explain the big change in growth rates and yields in presence of streptomycin. Flux variability analysis showed reactions that have flux category change in resistant strains compared to WT as a function of antibiotic involved redox cofactor balancing (NADH/NAD and NADPH/NADP ratios); increased TCA cycle/Oxidation Phosphorylation in the StrpR population and increased overflow metabolism population diverting from TCA cycle in the ChlR population. The wild type growth on pyruvate, malate and succinate also indicates the increase in NADH levels as a mechanism for survival in the presence of antibiotics (data not shown). The metabolism of these substrates after their uptake, all involve the use of the cofactor couple NAD/NADH [70]. The pareto front analysis of the added NADH/NADPH oxidase to the ChlR and StrpR strains potentially show an increase in NADH as a mechanism of evolution to become resistant. The addition of the above mentioned compounds potentially increase the NADH levels further causing major redox imbalance. This cofactor imbalance prevents the maintenance of a rigid core flux distribution, through certain control nodes, eventually preventing minimal cellular function of the metabolic network for energy and biomass formation. The model thus predicts the emergence of NAD/NADH ratios and electron imbalance to be critical to survival and susceptibility of the antibiotic resistant phenotype and can be leveraged to make the resistant pathogens susceptible to antibiotics again.

## Conclusion

Taken together, our data unveiled that disruption of redox homeostasis by certain benign metabolites as key to killing antibiotic resistant pathogens. To distinguish between causal and correlational factors in the evolution of antibiotic resistance more rigorous experiments and comprehensive genome-scale models may be needed. Reversing the phenotype by perturbing metabolism through modulating micro-environments could be one way of subverting the onset of the post antibiotic era. Determining the genetic and metabolic basis of ribosome targeting antibiotic’s resistance helps address the ‘fourth dimension’ of how heterogeneous networks in cells evolve simultaneously in space and time. This could lead to scalable pipelines integrating growth/metabolite/MIC profiling and constraints-based flux balance models for clinical isolates, ultimately leading to personalized treatment and individualized therapy.

## Additional files

Additional file 1: Supplementary Tables S1 to S6 and Figures S1 to S5. (PDF 2833 kb)

Additional file 2: Growth profiles for WT, ChlR and StrpR using 30 different C/N substrates when antibiotic was added from zero hour timepoint showing WT with no antibiotic in media (blue, wt_noab), ChlR with chloramphenicol added to the media (red, DD001_t0chl), StrpR with streptomycin added to the media (green, DD006_t0strep). Plots made using GraphPad Prism v6.01 and n = 3. (TIF 1004 kb)

Additional file 3: Growth profiles for WT, ChlR and StrpR using 30 different C/N substrates for first six hours when no antibiotic was added (antibiotic was added after these 6 h, and hence represented as ‘t6’) showing WT (blue) with no antibiotic in first 6 h, ChlR (DD001_t6chl, red), StrpR (DD006_t0strep, green) with no antibiotic in media. Plots made using GraphPad Prism v6.01 and n = 3. (TIF 778 kb)

Additional file 4: Growth profiles for WT, ChlR and StrpR using 30 different C/N substrates post addition of antibiotic at 6 h (t6) showing WT with chloramphenicol and streptomycin, (WT_t6chl,blue, and WT_t6strep, red, respectively), ChlR with chloramphenicol added to the media (DD001_t6chl,green), StrpR with with streptomycin added to the media (DD006_t0strep, violet). Plots made using GraphPad Prism v6.01 and n = 3. (TIF 1060 kb)

Additional file 5: This file contains information about the *C. violaceum* core metabolic model. The file includes metabolites, reactions, and other simulations used to understand the in silico behaviour of redox state of the resistant populations ChlR and StrpR in comparison to WT in the subsequent tabs. (XLS 384 kb)

Additional file 6: This file contains information about 59 metabolites analyzed using HPLC-HESI-HRMS. The file includes information including the retention time information, QC data peak area, normalized peak area among others. (XLS 292 kb)

## Abbreviations

2,5-DHB: 2,5-dihydroxybenzoic acid
2OXOADP: Oxoadipic acid
3PG: 3-phosphoglyceric acid
ACALD: Acetaldehyde dehydrogenase (acetylating)
AGC: Automated gain control
AKGDH: 2-Oxogluterate dehydrogenase
ALA: Alanine
ALCD2x: Alcohol dehydrogenase (ethanol)
ALE: Adaptive laboratory evolutions
AM-XIC: Accurate mass - extracted ion chromatogram
AP-MALDI: Atmospheric Pressure - Matrix-assisted Laser Desorption/ionization
ARA: L-Arabinose
ASCB: Ascorbic acid
ASP: Aspartate
ATCC: American type culture collection
ATP: Adenosine triphosphate
ATP4Sr: ATP synthase (four protons for one ATP)
ATV: Atorvastatin
BP: Base pairs
CAT: Category
CFU/ML: Colony-forming units per milliliter
CHCA: 2-cyano-4-hydroxycinnamic acid
CHL: Chloramphenicol
ChlR: Chloramphenicol resistant population of *Chromobacterium violaceum*
CIT: Citric acid
COBRA: Constraints based reconstruction and analysis
D-MAL: D-Malic acid
DNA: Deoxyribose nucleic acid
EX_ETOH(e): Ethanol exchange
FBA: Flux Balance Analysis
FDP: Fructose 1,6-bisphosphate
FORTi: Formate transport via diffusion
FUM: Fumarate
FVA: Flux variability analysis
FWHM: Full width at half maximum
G3P: Glyceraldehyde-3-phosphate
G6P: Glucose 6-phosphate
GDW: Grams dry weight
GLC: Glucose
GLN: Glutamine
GLU: Glutamate
GLYC: Glycerol
HESI: Heated electrospray ion source
HPLC: High performance liquid chromatography
HRMS: High-resolution mass spectrometry
ICIT: Isocitric acid
KGA: Ketoglutaric acid
LACT: L-Lactic acid
LB: Luria Bertani
LBA: Luria Bertani Agar
LC-MS: Liquid chromatography–mass spectrometry
LP: Linear Programming
M/Z: Mass by charge ratio
M6P: Manose-6-phosphate
MAL: Maleate or Maleic acid
MAN: Mannitol
MDH: Malate dehydrogenase
MEW: Mass extraction window
MIC: Minimum inhibitory concentration
MLO: Malonic acid
MS/MS: Tandem mass spectrometry
NAD+: Nicotinamide adenine dinucleotide (oxidised form)
NADH: Nicotinamide adenine dinucleotide (reduced form)
NADP+: Nicotinamide adenine dinucleotide phosphate (oxidised form)
NADPH: Nicotinamide adenine dinucleotide phosphate (reduced form)
NADTRHD: NAD transhydrogenase
NGS: Next generation sequencing
OD_600_: Optical Density at 600 nanometre
OXA: Oxalic acid
PCR: Polymerase chain reaction
PDF: Pulse dynamic focussing
PDH: Pyruvate dehydrogenase
PFL: Pyruvate formate lyase
PIt2r: Phosphate reversible transport via symport
PPC: Phosphoenolpyruvate carboxylase
PYR: Pyruvate or Pyruvic acid
QC: Quality control
R5P: Ribose 5-phosphate
Sbt: D-Sorbitol
Strp: Streptomycin
StrpR: Streptomycin resistant population of *Chromobacterium violaceum*
SUCC: Succinate or Succinic acid
SUCOAS: Succinyl-CoA synthetase (ADP-forming)
TCA: Tricarboxylic acid Cycle
TCS: Two-component system
THD2: NAD(P) transhydrogenase
TRP: Tryptophan
VAL: Valine
WT: Wild Type *Chromobacterium violaceum*
ZIC-HILIC: Zwitterionic hydrophillic interaction Liquid Chromatography.
