# Supplementary material for: A scalable metabolite supplementation strategy against antibiotic resistant pathogen Chromobacterium violaceum induced by NAD+/NADH+ imbalance
Source: BMC Syst Biol. 2017 Apr 26;11:51. doi: 10.1186/s12918-017-0427-z (PMC5405553; doi:10.1186/s12918-017-0427-z)
Supplement: Supplementary file 1 — Supplementary Tables S1 to S6 and Figures S1 to S5. (PDF 2833 kb) [file 12918_2017_427_MOESM1_ESM.pdf]

Additional file 1: Supplementary Tables and Figures

A Scalable Metabolite Supplementation Strategy Against Antibiotic Resistant Pathogen *Chromobacterium violaceum* Induced By NAD+/NADH+ Imbalance

Deepanwita Banerjee, Dharmeshkumar Parmar, Nivedita Bhattacharya, Avinash Ghanate, Venkateswarlu Panchagnula and Anu Raghunathan*

Chemical Engineering Division, CSIR-National Chemical Laboratory, Dr. Homi Bhabha Road, Pune 411008, India

*** Corresponding Author, Email: anu.raghunathan@ncl.res.in**

Samples	Total base reads	Mapped reads	Mean length	Average Base coverage depth (%)	Genome base coverage (%)		
					1x	20x	100x
ChIR	280780471	1565267	180bp	59.10	96.16	77.07	18.61
StrpR	229212916	1223568	188bp	48.24	95.3	71.30	10.01

Table S1: Coverage details about next generation sequencing using Ion torrent^{TM} Platform

Abbreviations used: bp – Base pairs

	Metabolite	Abr	MW	Concentration (mg/mL)	mM in media
1	Glucose	Glc	180.16	2	5.55
2	Glucose 6-phosphate	g6p	260.14	2	3.84
3	Glyceraldehyde-3-phosphate	g3p	170.06	2	5.88
4	Fructose 1,6-bisphosphate	fdp	340.12	2	2.94
5	Fumarate	fum	116.07	2	8.62
6	Maleic acid	Mal	116.07	2	8.62
7	D-Malic acid	D-Mal	134.09	2	7.46
8	Succinate	succ	118.09	2	8.47
9	Oxalic acid	oxa	126.07	2	7.93
10	Oxoadipic acid	2oxoADP	160.12	2	6.25
11	Malonic acid	MLO	104.06	1	4.80
12	Pyruvate	pyr	88.06	2	11.36
13	Citric acid	cit	192.12	2	5.21
14	Isocitric Acid	icit	192.12	2	5.20
15	L-Lactic acid	lact	90.08	0.27	1.50
16	Ketoglutaric acid	kga	146.11	2	6.84
17	L-Arabinose	ara	150.13	2	6.66
18	Manose 6 Phosphate	m6p	260.03	2	3.85
19	Ribose 5-phosphate	r5p	230.11	2	4.35
20	3-phosphoglyceric acid	3pg	186.06	2	5.37
21	L-Tryptophan	trp	204.23	2	4.90
22	L-Alanine	ala	89.09	2	11.22
23	L-Valine	val	117.15	2	8.54
24	L-Aspartate	asp	133.11	2	7.51
25	L-Glutamine	gln	146.15	2	6.84
26	L-Glutamate	glu	147.13	2	6.80
27	Mannitol	man	182.17	2	5.49
28	D-Sorbitol	sbt	182.17	2	5.49
29	Glycerol	glyc	92.09	2	10.86
30	L-Ascorbic acid	ascb	176.12	2	5.68

Table S2. List of Abbreviations for the 30 exogenous carbon and nitrogen sources, Related to Figure 3. Each row represents a substrate and the columns represent the metabolite name followed by abbreviation used, molecular weight (MW) and concentration in mg/mL and mM.

Time	Biomass (g/ml)	GR (hr-1)	Glucose (mM)	Violacein (mM)	G DW-HR	GUR	VSR	Molar Yield
**WT**								
**0**	0.00011384		0.2101	0.0032				
**6**	0.00167334	0.4480	0.0899	0.0079	0.0037	32.1892	2.1077	0.0139
**12**	0.00477787	0.3114	0.0569	0.0228	0.0153	9.9879	1.4887	0.0312
**18**	0.00405059	-0.0275	0.0808	0.0207	-0.1472	-0.8790	-0.1404	0.0313
**24**	0.00512718	0.0393	0.0686	0.0254	0.1305	1.0840	0.1946	0.0362
**30**	0.00528973	0.0052	0.0792	0.0177	1.0169	0.1287	0.0174	0.0404
**ChlR**								
**0**	0.00006847		0.2320	0.0016				
**6**	0.0008036	0.4105	0.1105	0.0027	0.0020	62.0272	1.3600	0.0066
**12**	0.00363592	0.3310	0.1163	0.0074	0.0110	10.5316	0.6730	0.0314
**18**	0.00410753	0.0203	0.0751	0.0440	0.2021	0.7761	0.2176	0.0262
**24**	0.00293519	-0.0560	0.0668	0.0387	-0.0524	-3.1508	-0.7378	0.0178
**30**	0.00238209	-0.0348	0.0557	0.0368	-0.0685	-2.5752	-0.5381	0.0135
**StrpR**								
**0**	9.1293E-05		0.1859	0.0037				
**6**	0.00047289	0.2741	0.0848	0.0027	0.0017	58.5722	1.5937	0.0047
**12**	0.00288381	0.3013	0.0636	0.0067	0.0096	12.7773	0.7021	0.0236
**18**	0.00496324	0.0724	0.0874	0.0179	0.0685	1.4376	0.2609	0.0504
**24**	0.0041377	-0.0123	0.0851	0.0261	-0.3377	-0.2985	-0.0773	0.0410
**30**	0.00404892	-0.0036	0.0922	0.0246	-1.1199	-0.0837	-0.0220	0.0432

Table S3 Table showing calculations for estimation of Glucose uptake rate (GUR), violacein secretion rates (VSR) and molar yield of Biomass among the three strains which were used as constraints in the *in silico* models.

A

	Category Description	Min	Max	Flexible/Rigid
1	POSITIVE FIXED	+a	+a	Rigid
2	POSITIVE VARIABLE	+b	+a	Flexible
3	ZERO TO POSITIVE	0	+a	Flexible
4	NEGATIVE FIXED	-a	-a	Rigid
5	NEGATIVE VARIABLE	-a	-b	Flexible
6	NEGATIVE TO ZERO	-a	0	Flexible
7	NEGLIGIBLE FLUX	small	small	Flexible
8	REVERSIBLE	-a	+a	Flexible
9	BLOCKED	0	0	Rigid

*a>b >0, -0.001 > small < 0.001

B

	Glucose		
γ redox(mets)	WT	ChlR	StrpR
nad[c]	0	0.0014	-0.0031
nadh[c]	0	0	0
nadp[c]	-0.0054	-0.0041	-0.0080
nadph[c]	0	0	0
γ redox (nadh/nad)	0	-0.0014	0.0031
γ redox (nadph/nadp)	0.0054	0.0041	0.0080
	just right	limiting redox	excess redox

Table S4 a Flux variability analysis (FVA) categorization based on flux through each reaction in the model. **b** The table shows γ_{redox}, calculated from the sensitivity analysis as a solution vector for the corresponding dual problem of FBA. This is an index of available reducing capacity available to the cell and whether it is limiting or in excess for biomass formation. γ_{redox} (nadh/nad) and γ_{redox} (nadph/nadp) are representative of reducing capacity available for ATP generation and biosynthetic reducing equivalent respectively. **ChlR** and **StrpR** show negative and positive γ_{redox}(nadh/nad) indicating the need for proper electron balance for optimal growth. The shadow price for wildtype is 0. A positive shadow price suggests available reducing capacity in excess of the optimal demand for growth and where it is negative, limiting for optimal growth. Metabolic reprogramming is essential to balance redox metabolism. If this does not happen, the cell can die as when the limiting carbon source is malate, succinate and pyruvate.

S.NO	Sequence Name	Sequence	No.Of Bases	Tm	GC%	amplicon size (base pairs)
1	0066_FW	CTGTTCCTCCCGCTCCG	17	59.44	70.59	452
2	0066_REV	TCAACGCCGACACGCTG	17	60.73	64.71	
3	0112_FW	TGATTGATGTCGCGCCGTAT	20	60.25	50	489
4	0112_REV	GGTAGTCGAGGCGATGAAGG	20	59.97	60	
5	0189_FW	CATTATCCCCGATGGCGCTT	20	60.61	55	431
6	0189_REV	CAAGAACGTGGCGGAAGTG	19	59.43	57.89	
7	0436_FW	GTTCAGCGAGCAGGGC	16	57.92	68.75	428
8	0436_REV	CCACCGCCACTTCGTCC	17	60.42	70.59	
9	0464_FW	GGACGGCAAGACCAGCAATA	20	60.39	55	427
10	0464_REV	GTAGGTGGTGGCGTTGAGAG	20	60.39	60	
11	nc1_FW	CGGAAGTGAAGAGCCTGGTG	20	60.67	60	427
12	nc1_REV	AAGTGCCGTTGCCATCCTT	19	60.23	52.63	
13	0570_FW	GAGGTCAGGTTGGATGCGAT	20	59.82	55	567
14	0570_REV	GCAGGATACGGGACAGGAAA	20	59.46	55	
15	nc2_FW	GACGGGATGTTGCGGACTAT	20	59.9	55	536
16	nc2_REV	CATCGTGGTGGGACTCTTGG	20	60.39	60	
17	0740_FW	TCGAAGGAGAAATCGACGCC	20	60.18	55	386
18	0740_REV	CACTACATGGGCACCTCCAC	20	60.39	60	
19	0877_FW	CGCTGGAAATGACCGACGTG	20	61.96	60	410
20	0877_REV	CATCATCTGGCGGTAGTCCC	20	59.97	60	
21	nc3nd4_FW	CGTGAAAGGGCGGTGTTCTA	20	60.32	55	477
22	nc3nd4_REV	CGATGAAGGGCGGAATGGG	19	60.89	63.16	
23	1080_FW	GCCCGAGACCAAGGACAAG	19	60.38	63.16	329
24	1080_REV	CGGTATTCGCCACTTGCTTC	20	59.63	55	
25	nc5_FW	CAGAGCACCGAGTCCATCAA	20	59.75	55	301
26	nc5_REV	CCTCCTGGCTGGGAAACATC	20	60.39	60	
27	nc6_FW	GCGTCATTTCATCGTTCGGG	20	59.97	55	332
28	nc6_REV	GGCTGCCTTCGGAACAAAAC	20	60.32	55	
29	1199_FW	AACACGCTCTTGGGGATGG	19	60	57.89	372
30	1199_REV	TATCTCTTCCGTCAGCACGC	20	59.9	55	
31	1261_FW	CTGCTTGACCCGAGGCTAAT	20	59.82	55	516
32	1261_REV	GAAATTCTCGTCCAGGCGCT	20	60.74	55	
33	nc7_FW	CAGGGGACGGGGAGGAT	17	59.66	70.59	396
34	nc7_REV	CAGGCGATTGGTCAGGGAAA	20	60.32	55	
35	1296_FW	GGCAATGGCGAGGACTTC	18	58.5	61.11	403
36	1296_REV	TGCCAGATGATGGTTCCGAC	20	60.11	55	
37	1301_FW	TGGACAGTGGCAGCAAGG	18	59.89	61.11	488
38	1301_REV	AGTCGTGTAATAAGGCAGCAC	21	58.38	47.62	
39	nc8_FW	TGGTTAGAGCACCACCTTGAC	21	59.93	52.38	504
40	nc8_REV	GAACAGCTCCTTGACGGCAT	20	60.67	55	
41	1596_FW	CGCCTCGTCCTGTATGGA	18	58.48	61.11	492
42	1596_REV	GCCAAGGTGATGCTGTTCAT	20	58.82	50	
43	nc9_FW	CGTCCTCGATGGCTGTACG	19	60.3	63.16	486

44	nc9_REV	GTCTGCTCCAGCTCGGTATG	20	60.25	60	
45	1872_FW	TTGGGCGTGTTGATCTCTGG	20	60.32	55	419
46	1872_REV	ACCTCTGCTGTTCAAGACTCG	21	60	52.38	
47	1994_FW	TTCGTCTACCCGATGTTCCG	20	59.55	55	413
48	1994_REV	ACGCTCCACAGCCACATATC	20	60.18	55	
49	nc10_FW	TGCTTTCGGCATTCTTGTGG	20	59.4	50	512
50	nc10_REV	TTGAGCGAGGTCACTTTCCC	20	59.97	55	
51	2377_FW	CGAGGGGCTGGAAATCAG	18	57.45	61.11	405
52	2377_REV	TTAGTAGGCAGGGCGAAGTC	20	59.18	55	
53	2560_FW	AACGAGGAAACCGACGACAA	20	59.9	50	465
54	2560_REV	GAAGAACGAGTACCACGGCA	20	60.04	55	
55	nc11_FW	GCCATTTCAGGTGCGTCATC	20	59.9	55	471
56	nc11_REV	AATGCGCTGGCGAAGTTTTT	20	59.97	45	
57	2789_FW	CCGCCCTGCATCAACAAGTA	20	60.68	55	478
58	2789_REV	CCACGTAGTTCCACACCAGG	20	60.32	60	
59	nc13_FW	AGTTGATGCACGAAACAAAGC	21	58.27	42.86	512
60	nc13_REV	TTGTGGCAAGACCCTGCG	18	60.59	61.11	
61	nc14_FW	TATCAGCCACAGACCAAGCC	20	59.75	55	545
62	nc14_REV	GGTATCGGAAGAGGAGAGCG	20	59.41	60	
63	3076_FW	ACATACTGGCGGATCACTCG	20	59.61	55	498
64	3076_REV	CAACACCCACACGGTAATCG	20	59.21	55	
65	3101_FW	TGCCAGGACATCAATACCCG	20	59.82	55	383
66	3101_REV	TCCAGCACATAGGCGTGT	18	58.61	55.56	
67	3351_FW	TTACTCCCCGAAACCACAGA	20	58.28	50	294
68	3351_REV	CTGTTCAACGACGACCAGAT	20	57.93	50	
69	3458_FW	GAGCACGCCAACAATACCCT	20	60.68	55	554
70	3458_REV	CCTTCACCAGCAGCGAGTT	19	60.3	57.89	
71	nc15_FW	GCGATGTACCAGGCCAAGAT	20	60.18	55	463
72	nc15_REV	CAAGGAAGCGGACAGGAAGT	20	59.96	55	
73	3519_FW	GATCCGAACCGCCTCACC	18	60.2	66.67	453
74	3519_REV	GATGAATTGCTGGTTGGCCG	20	60.18	55	
75	3561_FW	GAGGTGGGTTCGACGATGAC	20	60.46	60	461
76	3561_REV	GGGTGTCTGCCTCGTAGATG	20	59.9	60	
77	nc16_FW	GGTCCCCCAGAAATACCTGC	20	60.11	60	475
78	nc16_REV	GGCTACCTGAACGGTGTTGA	20	59.97	55	
79	4028_FW	ATGGAGATGTGGGCTGCAAA	20	59.96	50	305
80	4028_REV	TCGTGGTGGTTCAAAGCGAT	20	60.25	50	
81	4102_FW	GCTCTGGAACACGGTATGC	19	58.62	57.89	563
82	4102_REV	GGAGGAAAGGCGGTGGATTT	20	60.32	55	
83	4129_FW	CTATGGTGTTCAGCACGAAGC	21	59.61	52.38	418
84	4129_REV	ACCTGAGTACCGAATGACCG	20	59.18	55	
85	4192_FW	CGGTCACTTCGTCGGTCT	18	59.05	61.11	415
86	4192_REV	ATCGCCGCCCAGTCCAT	17	61.19	64.71	
87	4384_FW	CGTGCGTGAAAAGCCGTATC	20	60.25	55	471
88	4384_REV	TTGTCGTCCATCTCGTCCAG	20	59.47	55	

89	4284_FW	CAGGAGCCTTACTTCGGCAA	20	60.04	55	404
90	4284_REV	AAGGTTTCGGTGTGGTCGTT	20	60.11	50	
91	0464b_FW	CAGCGTGTCGTCCTCGTATG	20	60.86	60	402
92	0464b_REV	CTGCTGGCGGTGGTGTC	17	60.74	70.59	
93	4010_FW	CGATACGCCTGAACCCATCA	20	59.9	55	485
94	4010_REV	CGCACTCCAGCAGATTGAAG	20	59.27	55	
95	nc17_FW	AGCTGACTTCACTGCCAAGC	20	60.89	55	416
96	nc17_REV	AGATCGACCCCAATCTGTGC	20	59.82	55	
97	nc18_FW	TCCTGTCGTGCTCGCTTG	18	60.05	61.11	402
98	nc18_REV	ATTCCAGCGTCCCAGCTTAC	20	60.11	55	
99	1137_FW	GTCGAGGATGTAACGGCTCC	20	60.25	60	421
100	1137_REV	GCAGGTCTTCTCGTCCTTGT	20	59.68	55	
101	0662_FW	ACTCCAACCACAAGAAAACCAC	22	59.24	45.45	402
102	0662_REV	GCCAGATTGACGACGGTG	18	58.53	61.11	
103	3410_FW	AAAGGCGAAGGCGTCCAGAT	20	62.19	55	305
104	3410_REV	GATCCGGCGATACAGGAAGG	20	60.04	60	
105	2590_FW	CTGCAATTCCTGGGGATGGA	20	59.74	55	409
106	2590_REV	GCATGAGCGGTAAGTACGGT	20	60.18	55	
107	1471_FW	GCTTCCGCTTCCAGATCCTT	20	60.11	55	420
108	1471_REV	CCTATCGCACTGAGCCTGTT	20	59.82	55	
109	1071_FW	GCTGGATTACCGCATGACCT	20	60.18	55	389
110	1071_REV	GCAGCGTGGTGAACTTGTC	19	59.72	57.89	
111	1144_FW	ATGTACCAGCCGTCCAGCTC	20	61.96	60	433
112	1144_REV	TGAACAACATCCAGAAAGGCG	21	59.12	47.62	
113	1962_FW	GTACTCGTCGCCCAACCTG	19	60.45	63.16	372
114	1962_REV	ACGTCCTCGCAGTGTTTCAG	20	60.6	55	
115	1063_FW	GGACGATGGAATATCCGACCA	21	59.38	52.38	415
116	1063_REV	ACATGCCGAGAAACTCCCTG	20	60.04	55	
117	nc19_FW	TTCGATGTACGTTGCTGTCA	20	57.57	45	583
118	nc19_REV	CAAGTTTGATGCTGGAGCGG	20	59.83	55	
119	nc20_FW	CGACGGCCTGTTGCAGA	17	60.01	64.71	369
120	nc20_REV	CTGGTCTACCTGTGCTACGC	20	60.18	60	
121	nc21_FW	ATCTGCGACGAACTGGGC	18	60.13	61.11	449
122	nc21_REV	GGGGTGTTCCTGCTGGTC	18	59.97	66.67	
123	4309_FW	CGGGTAAAAGCAAGCGGC	18	59.82	61.11	400
124	4309_REV	CTGGCGCTGACCGAGATG	18	60.58	66.67	
125	0962_FW	GGTTCCAGCCATTGCAGCTT	20	61.54	55	469
126	0962_REV	TTCAGACCAAGACCGCTCAG	20	59.68	55	
127	1470_FW	CGCTACTGGACAGCGTCAC	19	60.8	63.16	443
128	1470_REV	CAACGCCAAAACCGTCTCG	19	60.08	57.89	
129	4191_FW	GATTACTTGGGACGCTTGGC	20	59.27	55	424
130	4191_REV	TTTCTGGAGGCGTGTTGCTT	20	60.47	50	
131	0004nc22_FW	CGACGTGTAAGGGTGTAGGG	20	59.83	60	462
132	0004nc22_REV	GGGGTGCAGCTCTTTCTGAT	20	60.03	55	
133	0560_FW	CTTGACCGTGGAGCTGGAG	19	60.08	63.16	412

134	0560_REV	GCGACACCAAGACCTCGAT	19	59.78	57.89	
135	0772_FW	AGCTTTTCATCGCCTCCAGT	20	59.67	50	419
136	0772_REV	GCTGATTGCCCGTTTCAAGC	20	60.73	55	
137	0816_FW	CCACCGAGGACGAGCAGTA	19	61.04	63.16	400
138	0816_REV	GGGATTTCTATCATCGCGCC	20	58.92	55	
139	0821_FW	CGCCCGACATGGAGGAT	17	58.76	64.71	491
140	0821_REV	GCCAGCCTCGACTACGC	17	60.26	70.59	
141	nc23_FW	CGATTTCGTCCAAGGCTATCTG	22	59.2	50	411
142	nc23_REV	AAAGCGAACAAGCTGTCCCA	20	60.47	50	
143	1114_FW	CTTTCAGCTCCTCCACCAGC	20	60.68	60	415
144	1114_REV	AAGAATCGTGGGACGGCTAC	20	59.82	55	
145	nc24_FW	GTTGGCTTGATGCGGTTGC	19	60.73	57.89	440
146	nc24_REV	AAGTGGGAAGGCGAGTTGAAG	21	60.54	52.38	
147	nc25_FW	ACTACGGCTACTACATCGTGC	21	59.67	52.38	423
148	nc25_REV	CAAGGTGGACAGCTATTCCG	20	58.34	55	
149	1299_FW	TTCCGCCTTCATCTTCAGGA	20	58.72	50	407
150	1299_REV	GCCGCAAGTACACCTTCCTC	20	61.02	60	
151	0304_FW	GTAAGCTGGGACGCTGGAAT	20	60.11	55	990
152	0304_REV	CGGGATGGCCGTTTTTCTTT	20	59.4	50	
153	0700_FW	CAATCATGCCCCTCGCACTA	20	60.18	55	1272
154	0700_REV	GCCCAGTTTCGGCATTTTCA	20	59.68	55	
155	2729_FW	TCTCCTCCTCCCGTCAACTT	20	59.89	55	880
156	2729_REV	CTGAGCGGGCTGGAAAAATG	20	59.83	55	
157	1455_FW	GCCAGTCCAGATTGTTCGGA	20	60.04	55	607
158	1455_REV	GAGGAAAGGCGGAAAATCGC	20	59.9	55	
159	4365_FW	AAGCCTCTACAATCTGCGGG	20	59.82	55	722
160	4365_REV	TGTGGCAGAAATGGGGTTCG	20	60.89	55	

Table S5: List of primers used in this study

Abbreviations: Tm - Melting temperature; GC% - Percentage GC content; FW - Forward Primer; REV - Reverse Primer; ncX_FW - Non Coding Region Forward Primer numberX, ncX_REV - Non Coding Region Reverse Primer number X.

Helix		Remarks	Amino acids
1	N-terminal		7-27
2		HTH DNA-binding motif	34-41
3			45-51
4	C-terminal	Ligand-binding domain	a:55-65 b:69-80
5			85-102
6			104-115
7			122-151
8		Ligand-binding domain involved in repression	160-180
9			190-204

Table S6: AcrR protein domain with sequence change (in yellow) results in truncation of ligand binding C- terminal involved in dimer formation and repressor binding.

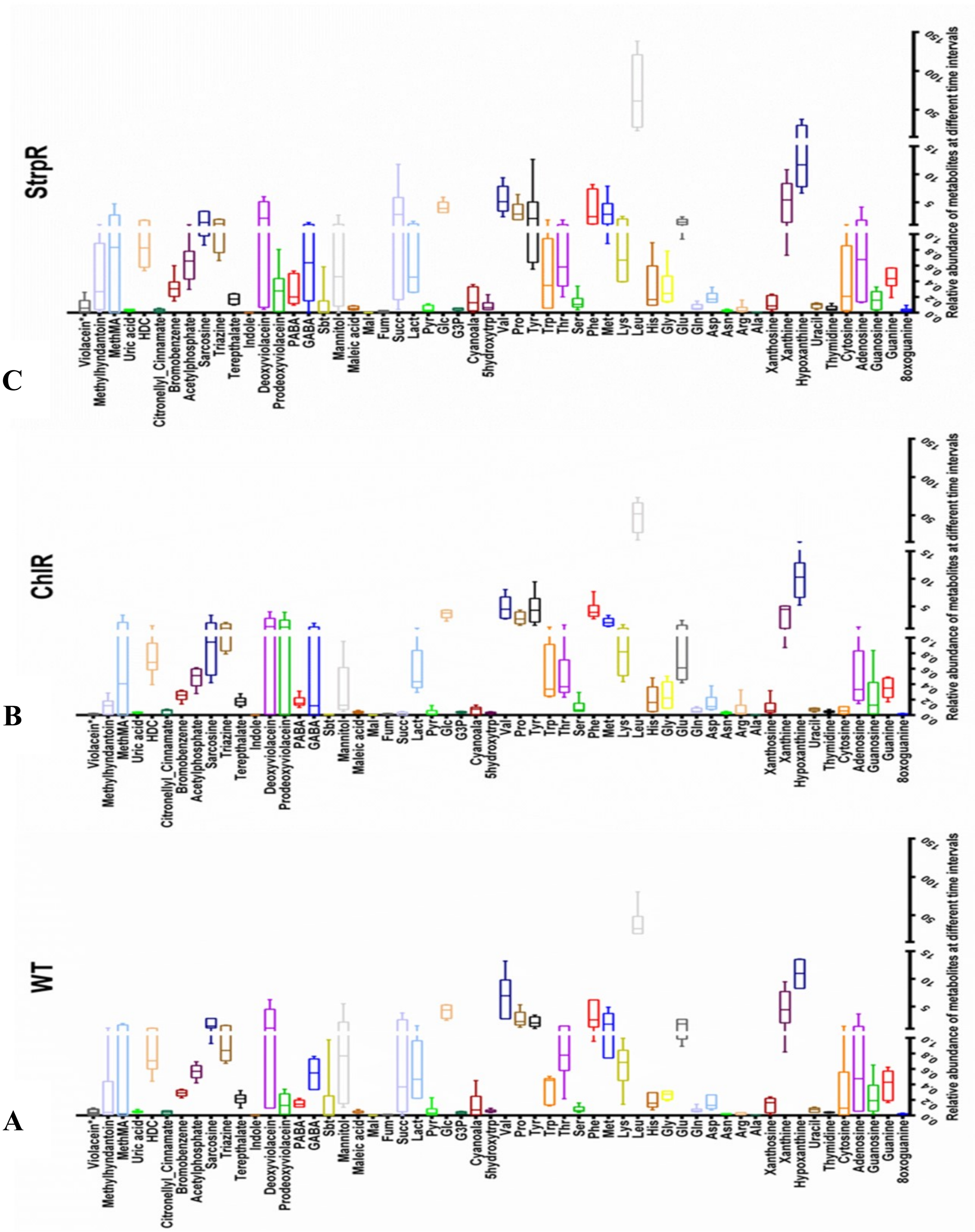

Figure S1 Dynamic metabolomic profiling across sensitive and resistant populations show metabolic reprogramming

Intracellular quantitative metabolite dynamics during growth on glucose of sensitive and resistant populations showing relative abundances ranging three orders of magnitude. All metabolites are color coded similarly across the three populations (**a** – WT, **b** – ChlR and **c** – StrpR) for easy comparison.

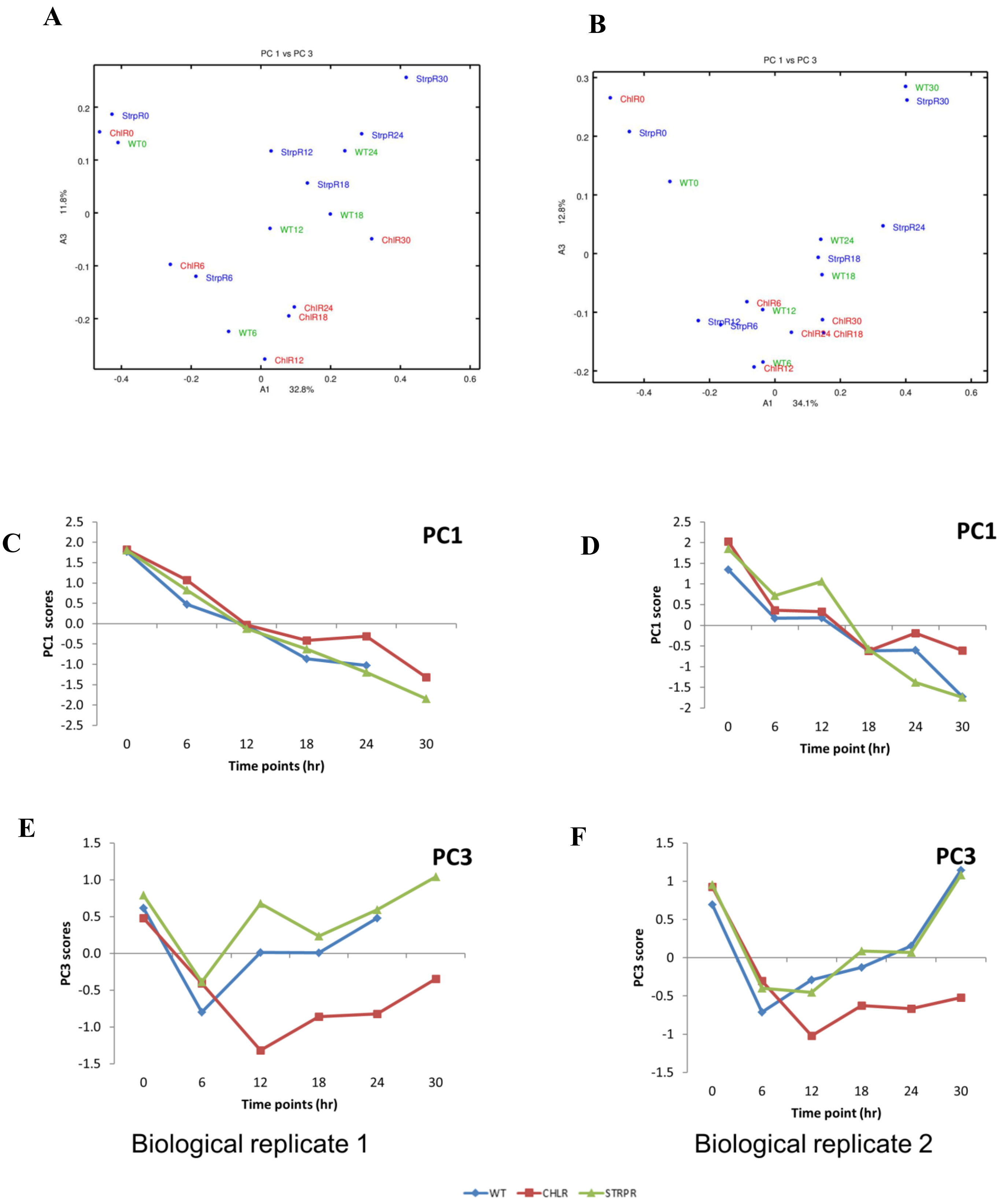

A
PC 1 vs PC 3
StrpR30
StrpR0
ChlR0
WT0
StrpR24
StrpR12
WT24
StrpR18
WT18
WT12
ChlR30
ChlR6
StrpR6
ChlR24
ChlR18
WT6
ChlR12
A1 32.8%
A3 11.8%
B
PC 1 vs PC 3
ChlR0
WT30
StrpR30
StrpR0
WT0
StrpR24
WT24
StrpR18
WT18
ChlR6
WT12
StrpR12
StrpR6
ChlR30
ChlR24
ChlR18
WT6
ChlR12
A1 34.1%
A3 12.8%
C
PC1
PC1 scores
Time points (hr)
D
PC1
PC1 score
Time point (hr)
E
PC3
PC3 scores
Time points (hr)
F
PC3
PC3 score
Time point (hr)
Biological replicate 1
Biological replicate 2
WT
CHLR
STRPR

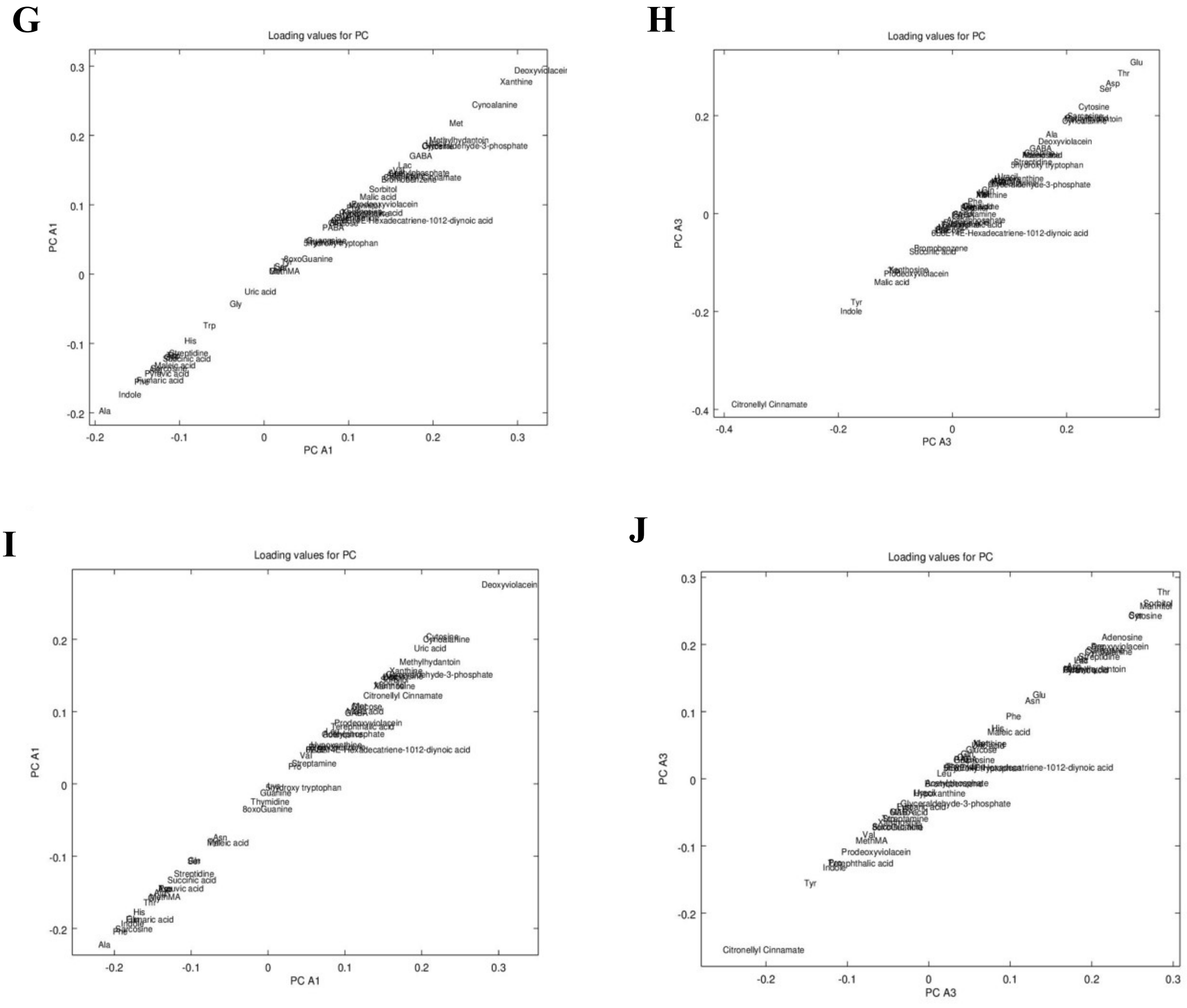

Figure S2 Principal Component Analysis (PCA) for LC-HRMS data
A plot of principle component 1 (PC1) versus 3 (PC3) of Biological replicate 1 (**a**) and Biological replicate 2 (**b**) revealed differential behavior of sample class C (ChlR) as opposed to A (WT) and E (StrpR). The sample class C (ChlR) stands out in the graphical space with respect to component 3. **c –f** PCA scores with respect to time points. Plots C and D represents PC1 scores for biological replicate 1 and 2 respectively. Similarly, E and F represents PC3 scores for the replicates in same order. **g - j** The plot for loading values of principal component PC 1 and PC3 for the biological replicates are represented. The plot showcases the loading values calculated for different metabolites while generating respective orthogonal Eigen vectors. Essentially, a loading value represent differential importance given to metabolites in order to transforms the data in PC space. Thus, higher loading value for a metabolite should infer a significant contribution in capturing the variance captured by respective PC. From score plot of PC1 (**c,d**) and PC3 (**e,f**) it is pertinent that PC1 captures time point based metabolic variations whereas PC3 shows segregation of ChlR strain from WT and StrpR strains for samples from 12hrs onwards. Hence, metabolites with higher loading values for PC1 represented in (**g,i**) (for both biological replicates) should corroborate higher variance across time points. Similarly, for PC3 metabolites with higher loading values should be differentially expressed specifically in ChlR strain (**h** and **j**).

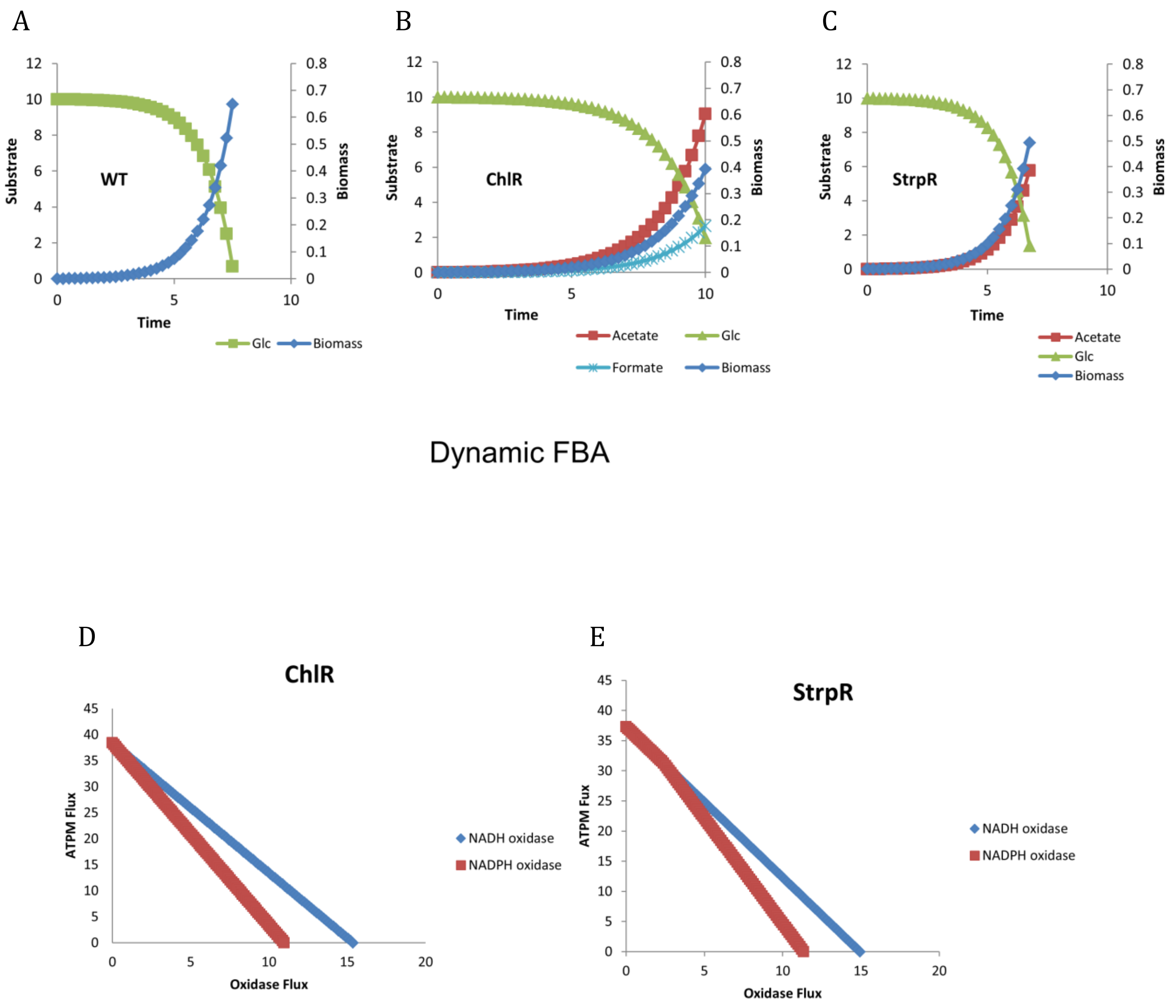

Figure S3 Additional simulations using iDB149.

a - c Dynamic FBA showing onset of Overflow metabolism. The predicted biomass concentration (secondary y – axis) and glucose, acetate, formate concentrations are plotted as a function of time for aerobic growth of different populations of *C. violaceum* WT, ChlR and StrpR respectively. The simulation was performed using experimental constraints (Refer Additional file 3) and initial concentration glucose as 10 mmol/g/hr. **d - e** Pareto front Trade-off between ATP, NADH and NADPH maintenance. The tradeoff associated with ATP maintenance versus NADH oxidase or NADPH oxidase was explored in order to understand the amount of NADH or NADPH dissipation necessary in order to match the experimental molar biomass yield of WT at the same ATP maintenance cost associated and similar oxygen uptake profiles.

A

Instrument	Wavelength	Slope	Intercept	R^2	Points	Range OD
Biophotometer	550	0.0209	-0.0002	0.9973	4	0.191 to 1.485
Microplate Reader	550	0.2161	-0.0713	0.9765	10	0.189 to 2.499

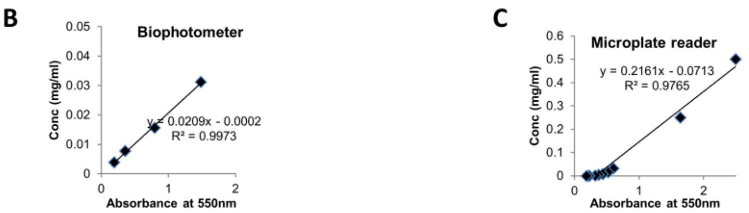

D

Instrument	g_{DW} (grams dry weight)
Biophotometer	0.0005*A650*1.25
Microplate Reader	0.0031*A650*1.19

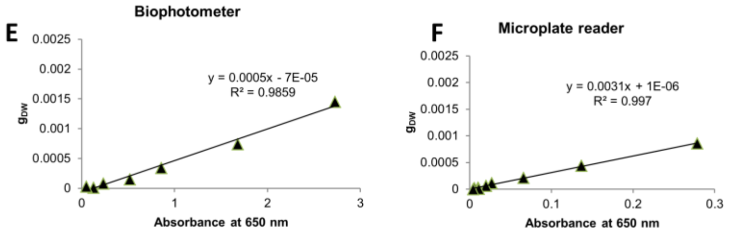

Figure S4 Calibration for Violacein and cell dry weight estimation
a - c Instrument specific violacein quantitation parameters followed by calibration plots for BioPhotometer (Eppendorf) and iMark™ Microplate absorbance reader (BIO-RAD) used in different experiments. **d - f** Instrument specific cell dry weight (g_{DW}) correction factors followed by calibration plots for the two instruments.

A

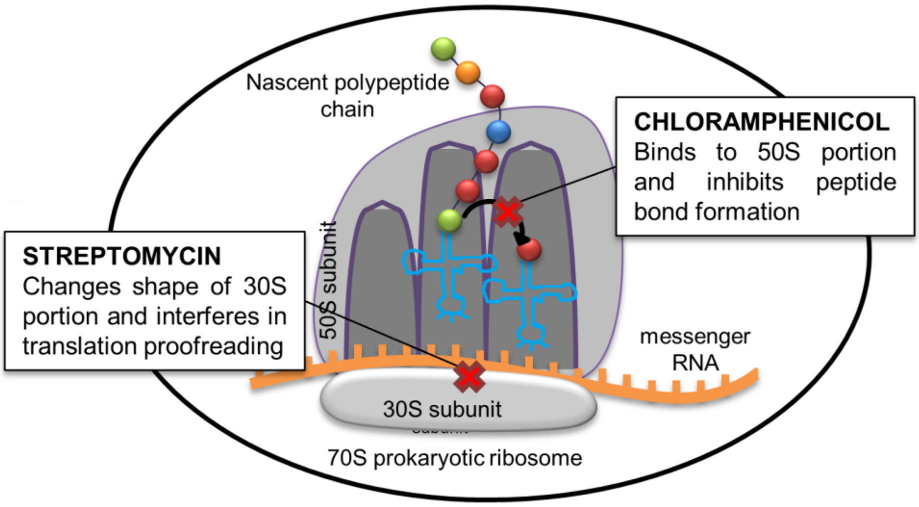
Nascent polypeptide chain
CHLORAMPHENICOL
Binds to 50S portion and inhibits peptide bond formation
STREPTOMYCIN
Changes shape of 30S portion and interferes in translation proofreading
50S subunit
messenger RNA
30S subunit
70S prokaryotic ribosome

B

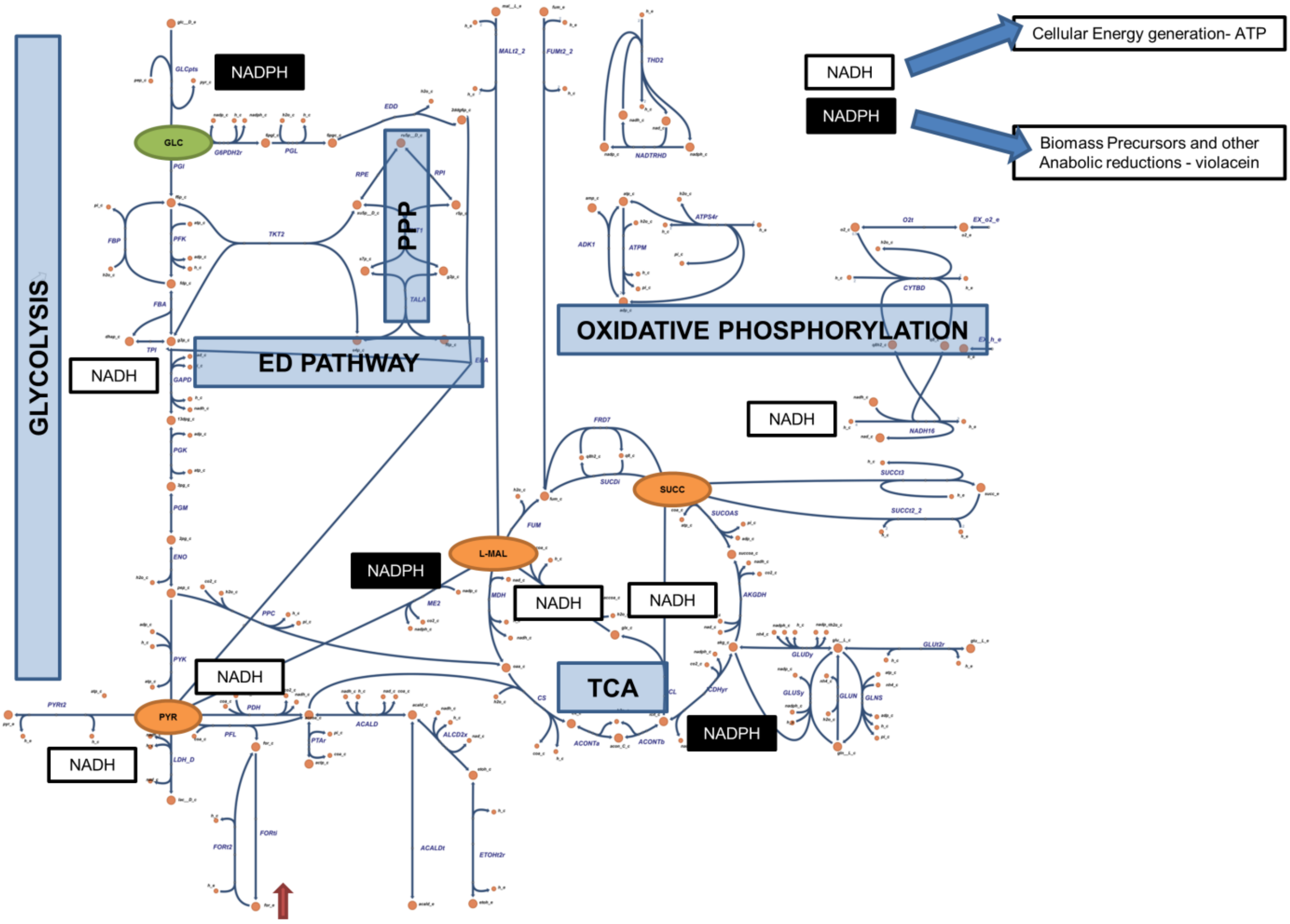
GLYCOLYSIS
NADPH
GLC
PPP
ED PATHWAY
NADH
OXIDATIVE PHOSPHORYLATION
SUCC
L-MAL
TCA
PYR
Cellular Energy generation- ATP
Biomass Precursors and other Anabolic reductions - violacein

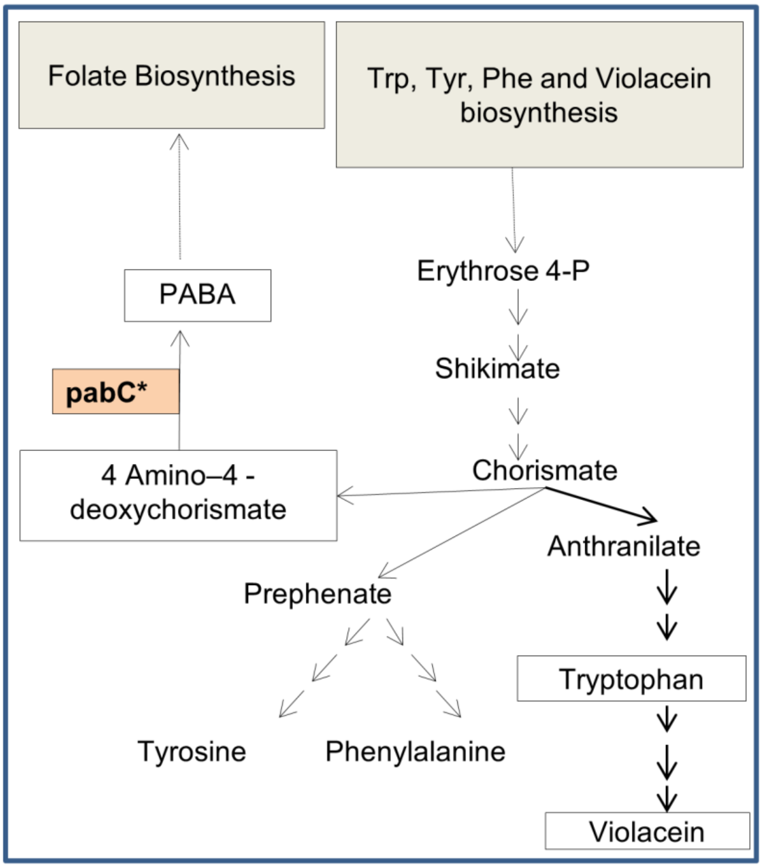

Figure S5 Various mechanisms of *C. violaceum* during antibiotic as a selection pressure.

a Schematic for explanation of mechanism of action of the two antibiotics on translational machinery. **b** Proposed mechanism of killing of *C. violaceum* as a result of cofactor and electron imbalance. Orange represents substrates which resensitize the resistant strains against the antibiotics and Green represents glucose, which is a happy condition for the cells. The model predicts the emergence of NAD/NADH ratios and electron imbalance to be critical to survival and susceptibility of the antibiotic resistant phenotype and can be leveraged to re-sensitise resistant pathogens to antibiotics. **c** *pabC* gene mutation potentially results in violacein increase only in StrpR population due to increase in flux through Tryptophan (Trp), Tyrosine (Tyr) and Phenylalanine (Phe) and violacein biosynthesis pathway.
