# Supplementary figures and images for: A scalable metabolite supplementation strategy against antibiotic resistant pathogen Chromobacterium violaceum induced by NAD+/NADH+ imbalance
Source: BMC Syst Biol. 2017 Apr 26;11:51. doi: 10.1186/s12918-017-0427-z (PMC5405553; doi:10.1186/s12918-017-0427-z)

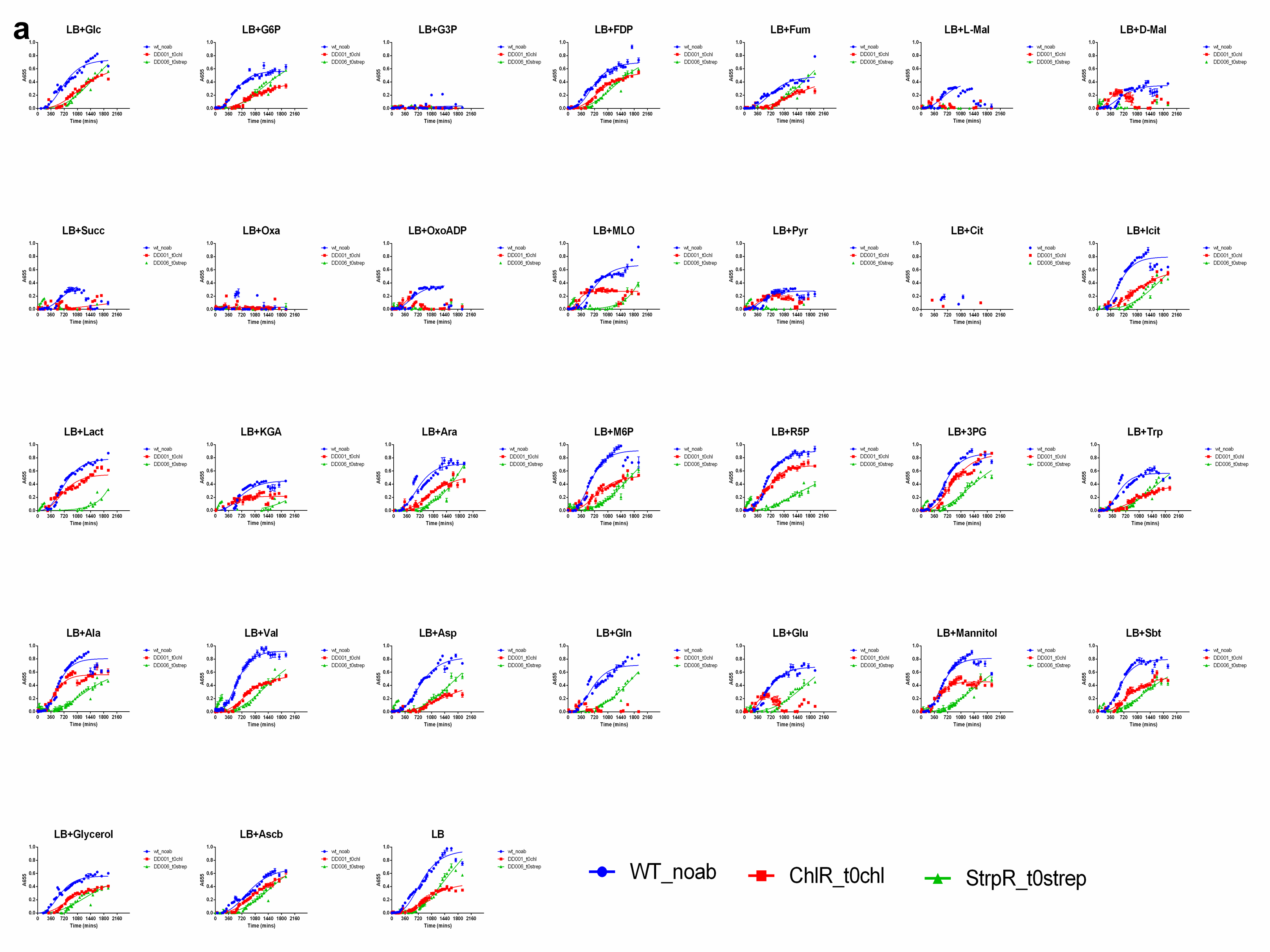

Supplement: Supplementary file 2 — Growth profiles for WT, ChlR and StrpR using 30 different C/N substrates when antibiotic was added from zero hour timepoint showing WT with no antibiotic in media (blue, wt_noab), ChlR with chloramphenicol added to the media (red, DD001_t0chl), StrpR with streptomycin added to the media (green, DD006_t0strep). Plots made using GraphPad Prism v6.01 and n = 3. (TIF 1004 kb) [file 12918_2017_427_MOESM2_ESM.tif]

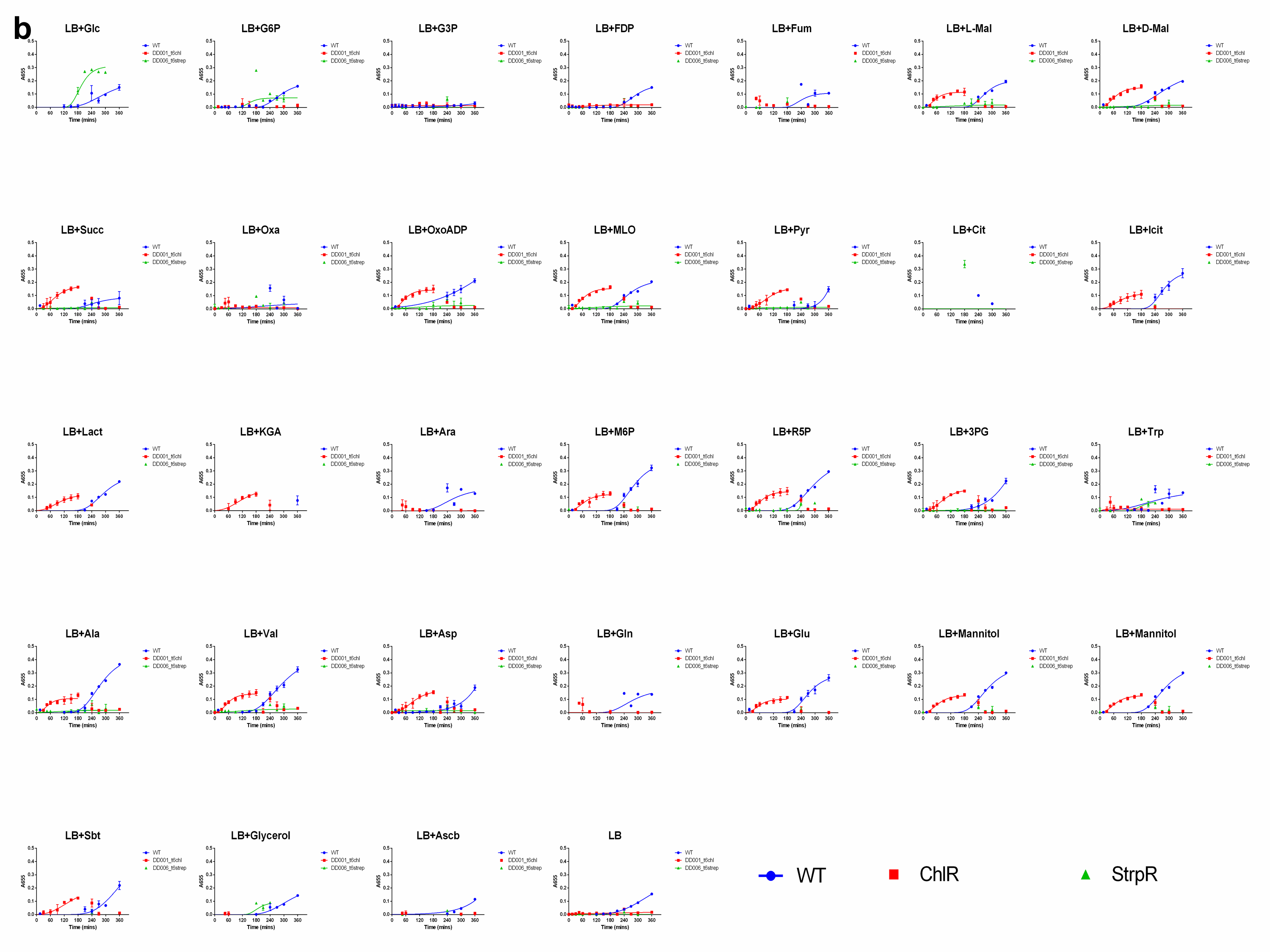

Supplement: Supplementary file 3 — Growth profiles for WT, ChlR and StrpR using 30 different C/N substrates for first six hours when no antibiotic was added (antibiotic was added after these 6 h, and hence represented as ‘t6’) showing WT (blue) with no antibiotic in first 6 h, ChlR (DD001_t6chl, red), StrpR (DD006_t0strep, green) with no antibiotic in media. Plots made using GraphPad Prism v6.01 and n = 3. (TIF 778 kb) [file 12918_2017_427_MOESM3_ESM.tif]

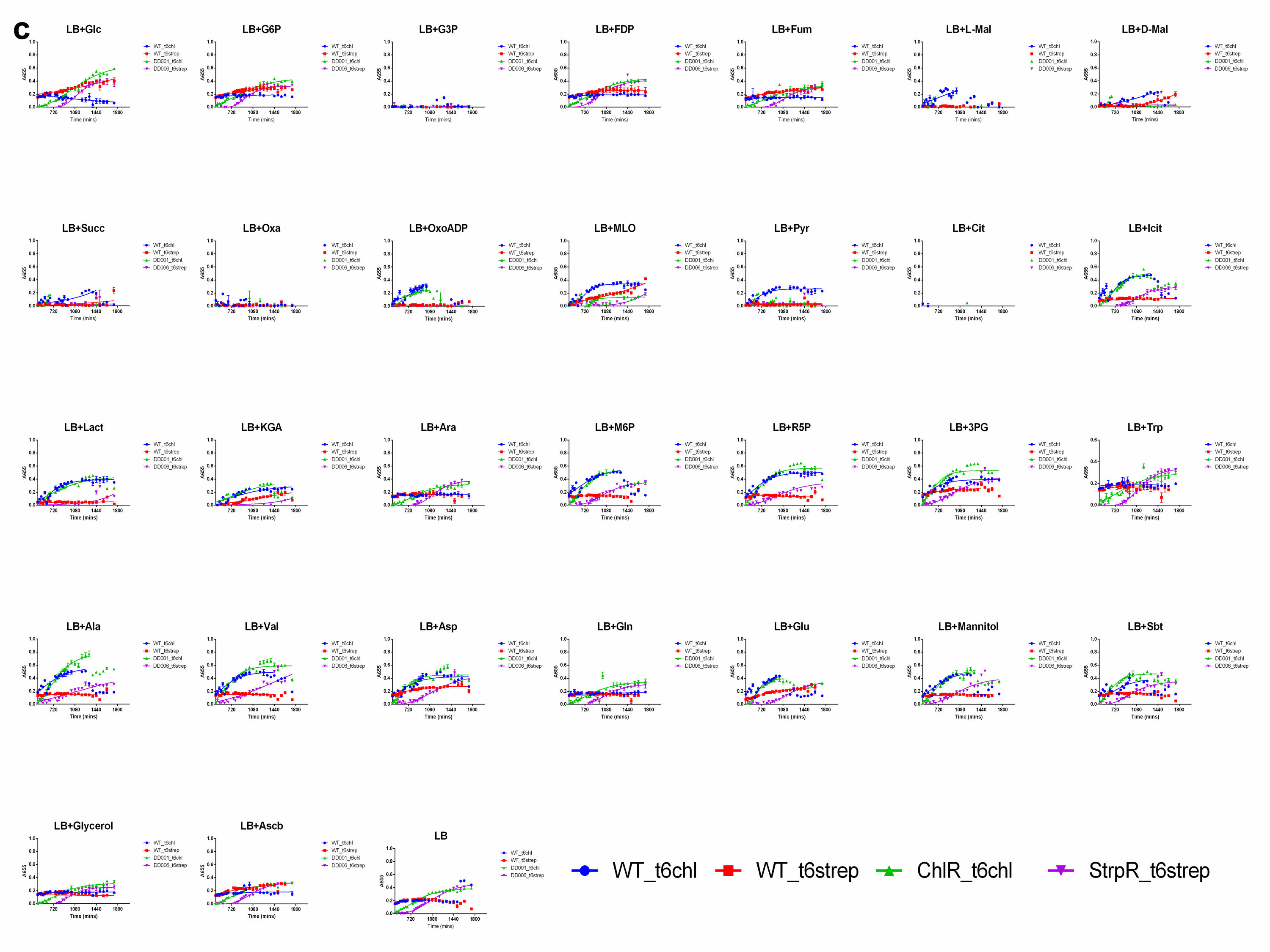

Supplement: Supplementary file 4 — Growth profiles for WT, ChlR and StrpR using 30 different C/N substrates post addition of antibiotic at 6 h (t6) showing WT with chloramphenicol and streptomycin, (WT_t6chl,blue, and WT_t6strep, red, respectively), ChlR with chloramphenicol added to the media (DD001_t6chl,green), StrpR with with streptomycin added to the media (DD006_t0strep, violet). Plots made using GraphPad Prism v6.01 and n = 3. (TIF 1060 kb) [file 12918_2017_427_MOESM4_ESM.tif]
